# Antiplatelet Treatment After Transient Ischemic Attack and Ischemic Stroke in Patients With Cerebral Microbleeds in 2 Large Cohorts and an Updated Systematic Review

**DOI:** 10.1161/STROKEAHA.117.020104

**Published:** 2018-05-10

**Authors:** Kui Kai Lau, Caroline E. Lovelock, Linxin Li, Michela Simoni, Sergei Gutnikov, Wilhelm Küker, Henry Ka Fung Mak, Peter M. Rothwell

**Affiliations:** 1 From the Centre for Prevention of Stroke and Dementia, Nuffield Department of Clinical Neurosciences, University of Oxford, United Kingdom (K.K.L., C.E.L., L.L., M.S., S.G., W.K., P.M.R.); 2 Division of Neurology, Department of Medicine (K.K.L.); 3 Department of Diagnostic Radiology (H.K.F.M.), Li Ka Shing Faculty of Medicine, University of Hong Kong.

**Keywords:** cerebral small vessel disease, magnetic resonance imaging, stroke, transient ischemic attack

## Abstract

**Background and Purpose—:**

In patients with transient ischemic attack/ischemic stroke, microbleed burden predicts intracerebral hemorrhage (ICH), and ischemic stroke, but implications for antiplatelet treatment are uncertain. Previous cohort studies have had insufficient follow-up to assess the time course of risks, have not stratified risks by antithrombotic use, and have not reported extracranial bleeds or functional outcome of ICH versus ischemic stroke.

**Methods—:**

In 2 independent prospective cohorts with transient ischemic attack/ischemic stroke (Oxford Vascular Study/mainly white; University of Hong Kong/mainly Chinese), antiplatelet treatment was started routinely irrespective of microbleed burden. Risks, time course and outcome of ICH, extracranial bleeds, and recurrent ischemic events were determined and stratified by microbleed burden (0 versus 1, 2–4, and ≥5), adjusting for age, sex, and vascular risk factors.

**Results—:**

Microbleeds were more frequent in the Chinese cohort (450 of 1003 versus 165 of 1080; *P*<0.0001), but risk associations were similar during 7433 patient-years of follow-up. Among 1811 patients on antiplatelet drugs, risk of major extracranial bleeds was unrelated to microbleed burden (*P*_trend_=0.87), but the 5-year risk of ICH was steeply related (*P*_trend_<0.0001), with 11 of 15 (73%) of ICH in 140 of 1811 (7.7%) patients with ≥5 microbleeds. However, risk of ischemic stroke also increased with microbleed burden (*P*_trend_=0.013), such that risk of ischemic stroke and coronary events exceeded ICH and major extracranial bleeds during the first year, even among patients with ≥5 microbleeds (11.6% versus 3.9%). However, this ratio changed over time, with risk of hemorrhage (11.2%) matching that of ischemic events (12.0%) after 1 year. Moreover, whereas the association between microbleed burden and risk of ischemic stroke was due mainly to nondisabling events (*P*_trend_=0.007), the association with ICH was accounted for (*P*_trend_<0.0001) by disabling/fatal events (≥5 microbleeds: 82% disabling/fatal ICH versus 40% disabling/fatal ischemic stroke; *P*=0.035).

**Conclusions—:**

In white and Chinese patients with ≥5 microbleeds, withholding antiplatelet drugs during the first year after transient ischemic attack/ischemic stroke may be inappropriate. However, the risk of ICH may outweigh any benefit thereafter.

## Abstract 2

Supplemental Digital Content is available in the text.

Cerebral microbleeds are markers of severe small vessel disease.^1–3^ Recent systematic reviews of mainly small studies have shown that a high burden of microbleeds is associated with an increased risk of intracerebral hemorrhage (ICH) and possibly also of ischemic stroke.^4–6^ Other retrospective studies have reported an increased risk of ICH among aspirin users with microbleeds.^7^ Although aspirin is highly effective in patients with transient ischemic attack (TIA)/ischemic stroke in reducing the early risk of recurrent ischemic events,^8^ and this benefit outweighs the risks of ICH,^9^ antiplatelet-related ICH is associated with a high risk of morbidity and mortality on longer-term treatment.^10^ However, in TIA/ischemic stroke patients with microbleeds, current guidelines make no recommendations on the safety of antiplatelet treatments^11^ although there is clinical uncertainty, particularly in those with ≥5 microbleeds.^12,13^ Clinicians, therefore, face a treatment dilemma^14^ and may err on the side of caution by not prescribing antiplatelets in patients with multiple microbleeds, potentially jeopardizing the early benefits of antiplatelet treatment after TIA/ischemic stroke.^8^

The treatment dilemma might be solved by reliable data on the prognostic implications of microbleeds, but the current evidence base has several shortcomings.^4–6^ First, meta-analyses have combined cohorts including patients who were variously on antiplatelets, anticoagulants, or no antithrombotic drugs.^5,6^ Second, although microbleeds are much more prevalent in Asians than whites, racial differences in prognosis remain uncertain. Third, although the balance of risk and benefits from antiplatelet agents in patients with microbleeds who present with a TIA/ischemic stroke might well vary over time, particularly if the risk of recurrent ischemic events is highest early whereas the risk of ICH and extracranial bleeding accrue more gradually, the time course of risk of ICH versus ischemic stroke has not been addressed previously. The particularly high early risk after TIA versus ischemic stroke might also be relevant here.^15^

To improve the reliability of previous estimates of risk of recurrent stroke from meta-analyses of small studies and to address these unanswered questions, we studied the time course and severity of recurrent events in TIA/ischemic stroke patients with microbleeds from OXVASC (Oxford Vascular Study), as well as a large prospective cohort of Chinese with ischemic stroke.

## Methods

Request for access to data will be considered by the corresponding author.

We prospectively studied 2156 patients with a probable or definite TIA/ischemic stroke recruited from 2 study centers—OXVASC and The University of Hong Kong (HKU). In brief, OXVASC is an on-going population-based study of all acute vascular events occurring within a predominantly white population of all 92 728 individuals, irrespective of age, who are registered with 100 general practitioners in 8 general practices of Oxfordshire, United Kingdom.^16^ The analysis herein includes 1080 consecutive cases of TIA/ischemic stroke recruited from November 2004 to September 2014 who had a cerebral magnetic resonance imaging (MRI) incorporating a hemosiderin-sensitive sequence and was subsequently diagnosed to have a TIA/ischemic stroke. The imaging protocol of OXVASC has been described in detail elsewhere.^17,18^ A further 1076 consecutive patients who were predominantly Chinese with a diagnosis of acute ischemic stroke who received an MRI scan incorporating a hemosiderin-sensitive sequence at the HKU MRI Unit was recruited from March 2008 to September 2014.^13^ Both cohorts had similar antiplatelet treatment policies, and antiplatelet treatment was started routinely irrespective of microbleed burden. However, patients with a diagnosis of cerebral amyloid angiopathy, defined according to the modified Boston criteria,^19^ presenting with a transient focal neurological episode,^20^ were not considered as having TIAs and were not included in this study.

All patients gave written informed consent, or assent was obtained from a relative of patients who were unable to provide consent. The 2 studies were approved by the local research ethics committee.

We collected demographic data, atherosclerotic risk factors, premorbid antithrombotic use, details of hospitalization of index event, and medications on discharge during face-to-face interview and cross-referenced these with primary care and hospital records in both cohorts.

Patients with TIA/ischemic stroke recruited from OXVASC were scanned with a 1.5-T or 3-T MRI scanner. All 1076 HKU patients were scanned using a 3-T MRI scanner. Microbleeds were detected using T2*-weighted gradient-recalled echo (GRE) in OXVASC and using susceptibility weighted imaging (SWI) in HKU. Details of scan parameters are provided in Table I in the online-only Data Supplement.

Two neurologists, supervised by 2 consultant neuroradiologists (H.K.F.M. and W.K.), interpreted all MRIs. Microbleeds were defined according to current guidelines,^21^ the location scored using the Microbleed Anatomical Rating Scale,^22^ and burden graded as absent, 1, 2 to 4, and ≥5.^6^ The intrarater κ for interpretation of microbleed burden in 50 randomly selected scans was 0.88 (OXVASC) and 0.81 (HKU), and the interrater κ was 0.84. White matter hyperintensity severity, enlarged perivascular space burden, and presence of lacunes were also determined based on previously validated scales (online-only Data Supplement).^23–25^

All patients in OXVASC were followed-up regularly by a research nurse or physician at 1, 3, 6, 12, 24, 60, and 120 months after the index event. Patients recruited from HKU were followed-up by a clinician every 3 to 6 months or more frequently if clinically indicated. All patients were assessed for the following clinical outcomes: (1) recurrent stroke (ischemic and hemorrhagic), (2) acute coronary events (acute coronary syndrome and sudden cardiac death), (3) major extracranial bleeding, and (4) mortality (vascular and nonvascular). The definition of recurrent stroke required a sudden new neurological deficit fitting the definition of ischemic stroke or ICH, occurring after a period of unequivocal neurological stability and not attributable to cerebral edema, mass effect, or hemorrhagic transformation of the incident cerebral infarction. Modified Rankin Scale (mRS) at 1 month after recurrent stroke was determined and disabling stroke defined as mRS >2 (refer to online-only Data Supplement for definitions of other clinical outcomes). Where needed, details of clinical outcomes were supplemented by medical records from primary care practices, hospitals, as well as the Deaths General Register Office.

We performed an updated systematic review according to the PRISMA guidelines (Preferred Reporting Items for Systematic Reviews and Meta-Analyses) and searched Medline and Embase from April 2015 (date last systematic review^6^ on this topic was performed) to September 2017 with the following search strategy:

1. Cerebral microbleed* or CMB or cerebral microh?emorr* or brain microbleed* or brain microh?emorr*
2. Stroke or isch?emic stroke or TIA or intrac* adj2 h?emorrhag* or ICH
3. 1 and 2

We included published and unpublished studies that fulfilled the following criteria: (1) included a study population of patients with TIA or ischemic stroke, (2) performed MRI T2*-GRE or SWI sequences at baseline to detect presence of microbleeds and had ischemic stroke or ICH as an outcome, (3) had a prospective study design with at least 3 months of follow-up, and (4) subjects were predominantly (≥70% of the study population) on antiplatelet agents.

### Statistical Analysis

We first conducted separate analyses for the OXVASC and HKU cohorts. Because there was no significant heterogeneity (microbleed burden by cohort interaction *P*=0.52 for prediction of recurrent stroke), these were then combined in a pooled analysis. The clinical and imaging predictors of ≥5 microbleeds were determined using logistic regression model. Variables, including age, male sex, vascular risk factors (hypertension, hyperlipidemia, diabetes mellitus, smoking, atrial fibrillation), glomerular filtration rate,^26^ premorbid use of antiplatelets, and anticoagulants, were entered into a univariate analysis model, and all variables were subsequently entered into a multivariate analysis model to determine the independent predictors of ≥5 microbleeds. The associations of ≥5 microbleeds with other neuroimaging markers of severe small vessel disease were also determined.

In the primary analysis, we used Kaplan–Meier survival analysis to calculate the 5-year risk of adverse events among 1811 antiplatelet users from OXVASC and HKU, censored at death or March 31, 2015. Risks of adverse events by microbleed burden were compared with log-rank test. We determined by Cox-regression analysis the unadjusted and adjusted (age, sex, and vascular risk factors) risks of adverse outcome among patients with 1, 2 to 4, and ≥5 microbleeds compared with no microbleeds as reference. We obtained the *P*_trend_ by analyzing 0, 1, 2 to 4, ≥5 microbleeds as a noncategorical variable. The following outcomes were studied: recurrent stroke, recurrent ischemic stroke, ICH, acute coronary events, major extracranial hemorrhage, death, and vascular death. We also examined the risk of adverse events of patients with <5 and ≥5 microbleeds, within 1 year versus those occurring from 1 to 5 years of index event using the Kaplan–Meier method. To determine whether the risk of recurrent ischemic stroke and ICH in patients with ≥5 microbleeds was time dependent, we inserted a time-dependent variable (within 1 year versus 1–5 years) into the Cox-regression model. Finally, we compared the severity of recurrent ischemic stroke and ICH based on mRS with ordinal regression (mRS shift) analysis.

Because there were no new studies from April 2015 to September 2017 that matched our systematic review search criteria (Figure I in the online-only Data Supplement), we performed a meta-analysis using a random-effects analysis by pooling the results from our 2 cohorts with those from the most recent systematic review^6^ and calculated the risk ratios of recurrent ischemic stroke and ICH among patients with 1, 2 to 4, and ≥5 microbleeds compared with no microbleeds as reference. TIA/ischemic stroke cohorts that were predominantly (≥70% of the study population) on antiplatelets were included. We stratified our analysis by ethnicity (whites versus Asians), MRI strength (0.5T/1T/1.5T versus 3T), and sequence (T2*GRE versus SWI). Heterogeneity was determined with χ^2^ tests.

Sensitivity analyses was also performed to include all 2083 patients, regardless of antithrombotic status, from OXVASC and HKU and all studies from the systematic review.^6^

All analyses were done with SPSS version 22.

### Role of the Funding Source

The funding source had no role in study design, data collection, data analysis, data interpretation, or writing of the report. The corresponding author had full access to all the data in the study and had the final responsibility for the decision to submit for publication.

## Results

After excluding 73 patients with incomplete follow-up data, a total of 2083 patients were included in the final analysis. Characteristics and outcomes of the 1080 patients from OXVASC (572 TIA, 508 ischemic stroke) and 1003 patients with ischemic stroke from HKU are shown in the Table. HKU patients were more often men and were more likely to have hypertension and diabetes mellitus whereas OXVASC patients were more likely to have hyperlipidemia or were ever-smokers (all *P*<0.05). The median delay from event to MRI was 3 days in OXVASC and 4 days in HKU. Cause of TIA/ischemic stroke, according to the modified TOAST criteria (Trial of ORG 10172 in Acute Stroke Treatment), is provided in Table II in the online-only Data Supplement. Antithrombotic treatment on discharge in the 2 cohorts was similar (Table), with 86.9% on antiplatelets only, 9.4% anticoagulants only, 1.3% anticoagulant plus antiplatelet, and 2.4% on no antithrombotic agents. There were no differences in microbleed burden in patients with or without antithrombotic treatment (Table III in the online-only Data Supplement; *P*=0.58).

**Table.**
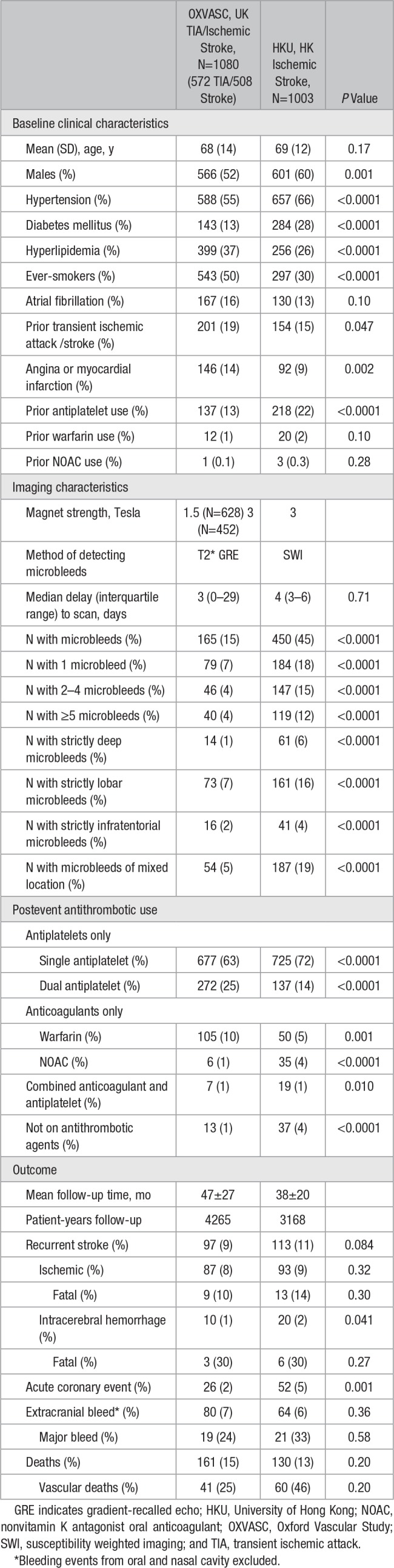
Characteristics and Outcomes of OXVASC and HKU Cohorts

Microbleeds were more frequent in the HKU cohort versus OXVASC (45% versus 15%; *P*<0.0001), but risk factors for microbleeds were similar (Table IV in the online-only Data Supplement). On multivariate analysis, only age (adjusted odds ratio, 1.02; 95% confidence interval, 1.00–1.03; *P*=0.035) and premorbid anticoagulation use (2.77; 1.00–7.70; *P*=0.05) remained significant independent predictors of a high burden (≥5) of microbleeds (Table IV in the online-only Data Supplement). Lacunes and an increasing burden of enlarged basal-ganglia perivascular spaces and white matter hyperintensity were all associated with ≥5 microbleeds after adjusting for age and sex (all *P*<0.05; Table V in the online-only Data Supplement).

On 4265 patient-years follow-up in OXVASC and 3168 patient-years in HKU, associations between microbleed burden and risk were similar in the 2 cohorts for all outcomes (Table VI in the online-only Data Supplement), and so further analyses are pooled. After mean follow-up of 43±25 months (7433 patient-years), 220 patients developed a recurrent stroke (82% ischemic; Table). One patient developed a subarachnoid hemorrhage because of an underlying cerebral aneurysm. Seventy-eight patients developed an acute coronary event and 144 patients an extracranial bleed. A total of 291 patients died during follow-up (34% vascular deaths).

**Figure 1.**
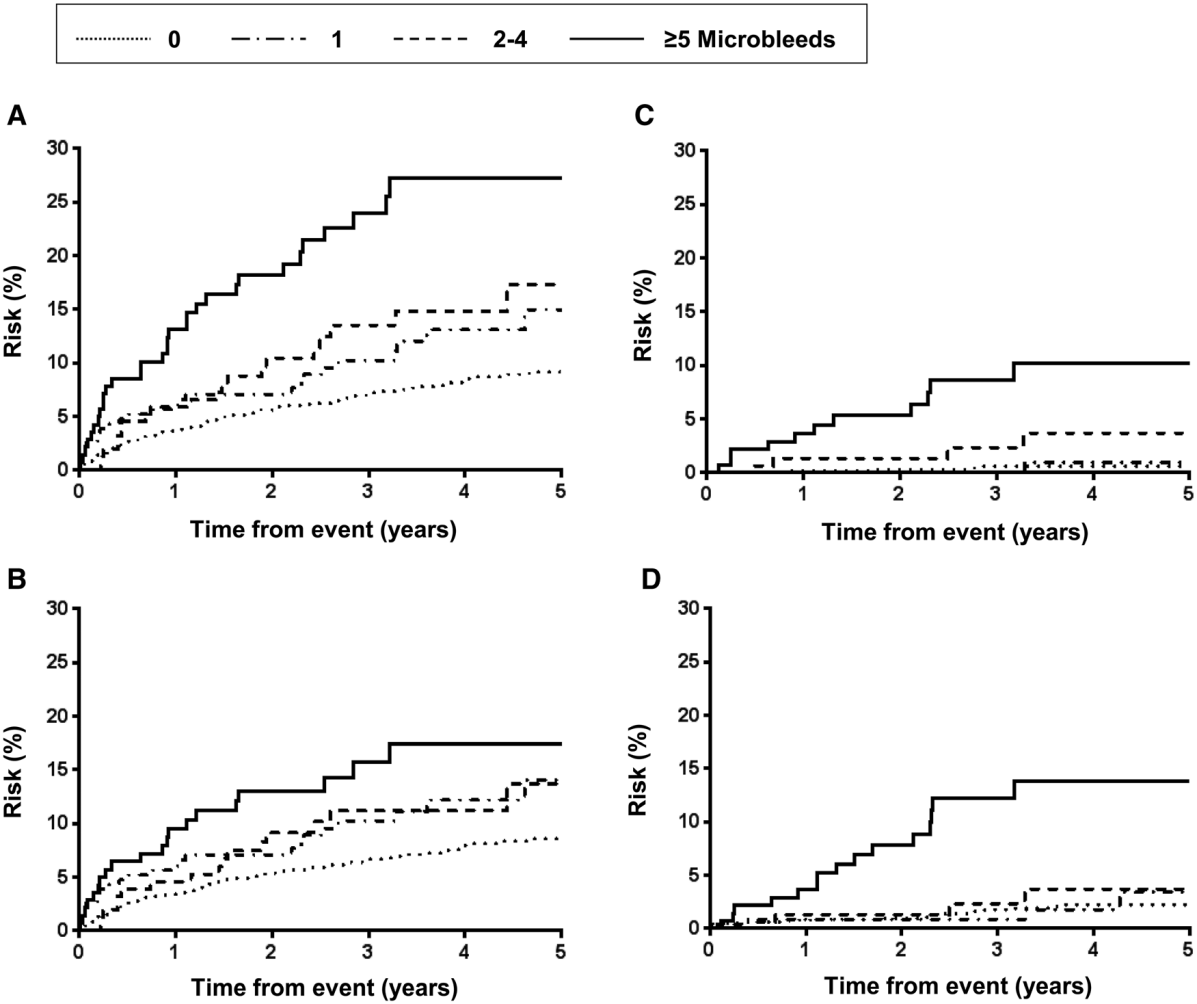
Risk of recurrent stroke (**A**), recurrent ischemic stroke (**B**), intracerebral hemorrhage (**C**), and intracerebral hemorrhage and major extracranial bleeding (**D**) among patients with transient ischemic attack/ischemic stroke on antiplatelets.

Among 1811 patients (OXVASC n=949; HKU n=862) who were prescribed with antiplatelet agents (26 with concomitant anticoagulant use excluded), the 5-year risks of recurrent ischemic stroke and ICH in patients with 0, 1, 2 to 4, and ≥5 microbleeds were 8.7%, 14.1%, 13.7%, and 17.4% (Figure 1B; log-rank test *P*=0.002) and 0.6%, 0.9%, 3.7%, and 10.2%, respectively (Figure 1C; *P*<0.0001). After adjusting for age, sex, and vascular risk factors, a high microbleed burden was an independent predictor of recurrent ischemic stroke, ICH, all-cause mortality, and nonvascular death (all *P*_trend_<0.05; Table VII in the online-only Data Supplement). A high microbleed burden was not associated with risk of coronary events, major extracranial bleed, or vascular death (all *P*_trend_>0.05). Similar findings were noted in 1403 patients (OXVASC n=677; HKU n=726) on single antiplatelets (Table VIII in the online-only Data Supplement).

**Figure 2.**
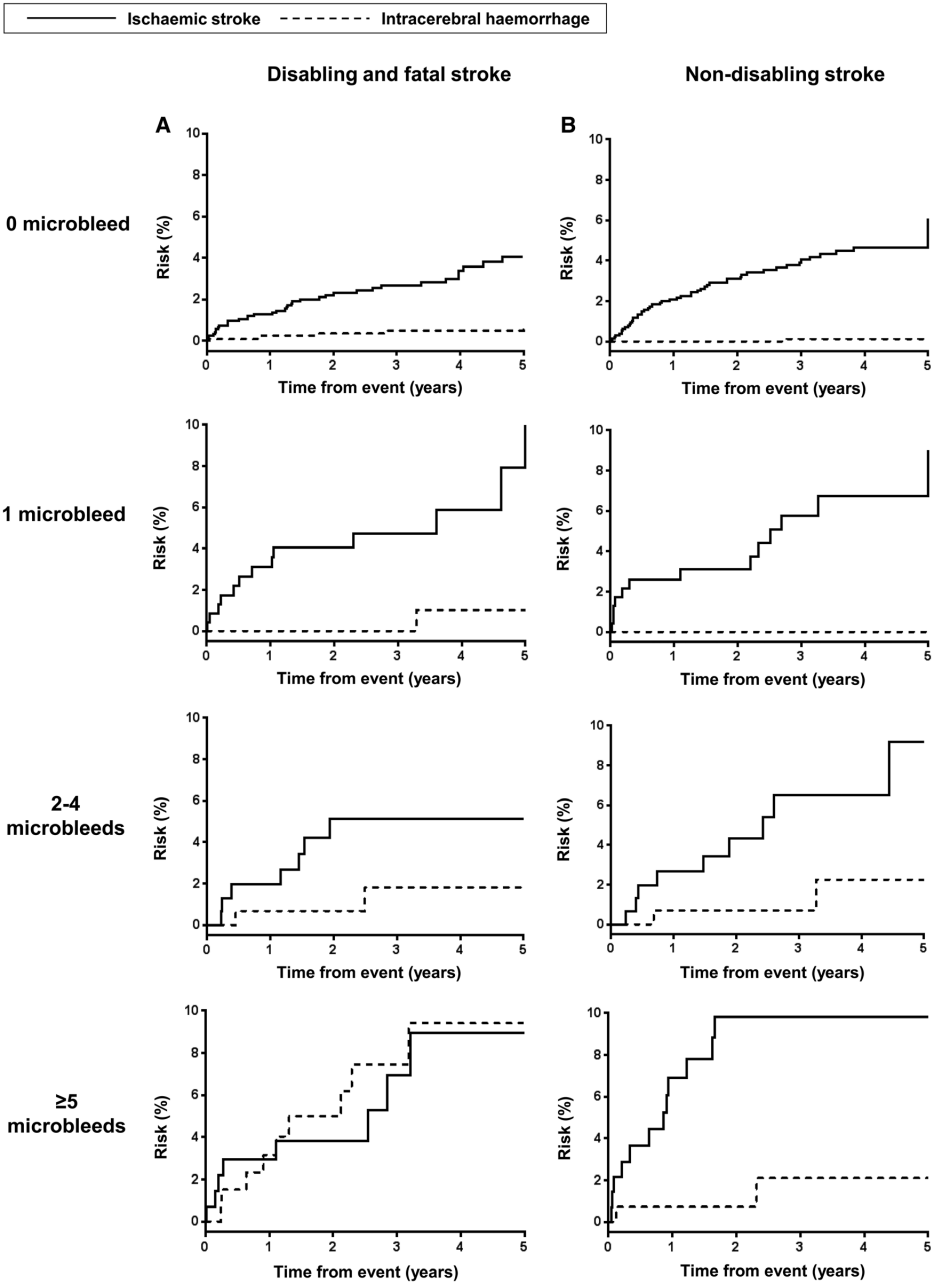
Risk of disabling or fatal (**A**) and nondisabling (**B**) ischemic stroke and intracerebral hemorrhage by microbleed burden in patients with transient ischemic attack/ischemic stroke on antiplatelets.

In patients with microbleeds, the 5-year absolute risks of a nondisabling ischemic stroke exceeded that of a nondisabling ICH (9.4% versus 1.2%; *P*<0.0001), even among those with ≥5 microbleeds (9.8% versus 2.1%; *P*=0.008; Figure 2). Similarly, the 5-year risk of a disabling/fatal ischemic stroke exceeded that of a disabling/fatal ICH in patients with 1 to 4 microbleeds (8.3% versus 1.3%; *P*=0.0004; Figure 2). However, in patients with ≥5 microbleeds, risks of a disabling/fatal ICH increased substantially, such that the 5-year absolute risks of a disabling/fatal ischemic stroke and ICH were similar (9.0% versus 9.4%; *P*=0.81; Figure 2). Moreover, in patients with ≥5 microbleeds, a greater proportion of patients who developed a subsequent ICH were disabled or dead compared with those who developed a recurrent ischemic stroke (81.8% versus 40.0%; mRS shift odds ratio, 6.75; 95% confidence interval, 1.14–39.80; *P*=0.035; Figure II in the online-only Data Supplement).

**Figure 3.**
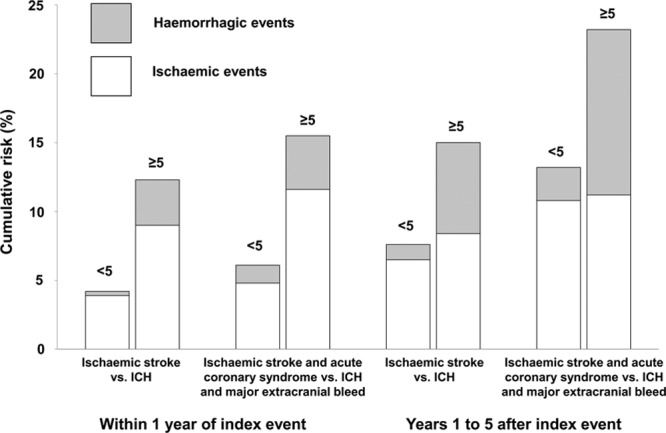
Risk of ischemic and hemorrhagic events in transient ischemic attack/ischemic stroke patients with <5 and ≥5 microbleeds on antiplatelets, within 1 year of index event and between 1 and 5 years after index event. ICH indicates intracerebral hemorrhage.

In a time-course analysis, among patients with <5 microbleeds, the absolute risks of ischemic stroke and coronary events combined exceeded that of an ICH and major extracranial bleed both within and beyond 1 year of the index event (1-year risk: 4.8% versus 1.3%; 1–5 year risk: 10.8% versus 2.4%; Figure 3). In patients with ≥5 microbleeds, risks of a combined ischemic event also exceeded that of a combined hemorrhagic event during the first year (11.6% versus 3.9%). However, in years 1 to 5, the risks of ICH increased steeply such that the risks of ICH matched that of ischemic stroke (11.2% versus 12.0%; Figure 3). This was mainly because of the expected frontloading of recurrent ischemic stroke risk during the first year after TIA/ischemic stroke as compared with years 1 to 5 (*P*=0.030). However, there was no evidence of time dependence of ICH risk in TIA/ischemic stroke patients with ≥5 microbleeds (*P*=0.74).

**Figure 4.**
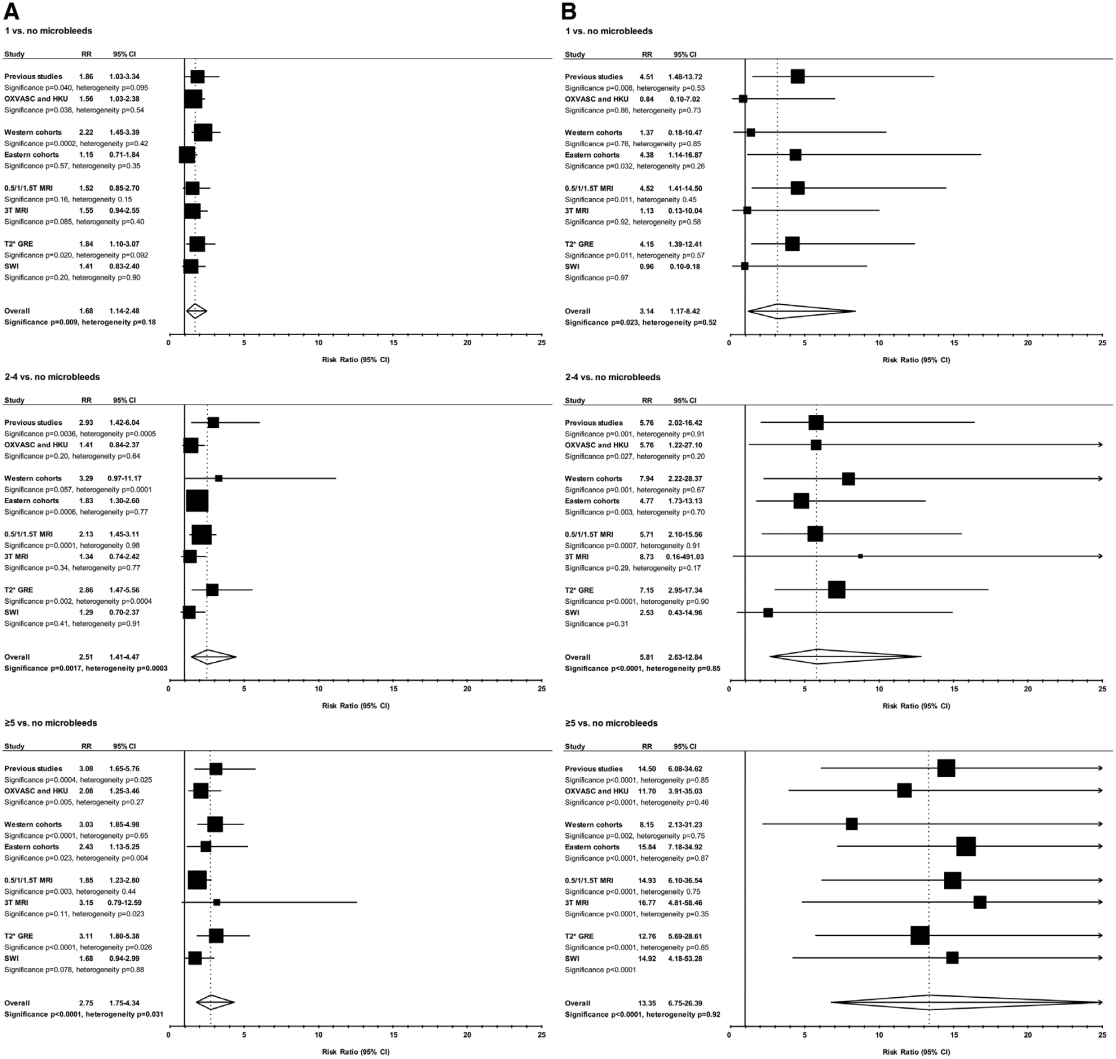
Pooled analyses of relative risk estimates from the current and previous studies showing risk of recurrent ischemic stroke (**A**) and of intracerebral hemorrhage (**B**) among patients with transient ischemic attack/ischemic stroke on antiplatelet agents with microbleeds vs those without, stratified by geographical origin, magnetic resonance imaging (MRI) scanner magnet strength, MRI sequence, and number of microbleeds. CI indicates confidence interval; GRE, gradient-recalled echo; HKU, University of Hong Kong; OXVASC, Oxford Vascular Study; RR, relative risk; and SWI, susceptibility weighted imaging.

We pooled the results from OXVASC and HKU with those from a recent meta-analyses (Table IX in the online-only Data Supplement).^6^ After excluding cohorts with <70% of the study population on antiplatelet agents, the pooled unadjusted relative risk estimates of recurrent ischemic stroke in patients with 1, 2 to 4, and ≥5 versus no microbleeds were 1.68 (95% confidence interval, 1.14–2.48; *P*=0.009; *P*_het_=0.18), 2.51 (1.41–4.47; *P*=0.002; *P*_het_=0.0003), and 2.75 (1.75–4.34; *P*<0.0001; *P*_het_=0.031; Figure III in the online-only Data Supplement). The pooled relative risk estimates of ICH in patients with 1, 2 to 4, and ≥5 versus no microbleeds were 3.14 (1.17–8.42; *P*=0.023; *P*_het_=0.52), 5.81 (2.63–12.84; *P*<0.0001; *P*_het_=0.85), and 13.35 (6.75–26.39; *P*<0.0001; *P*_het_=0.92; Figure IV in the online-only Data Supplement). No significant heterogeneity was noted between our 2 cohorts and pooled relative risk estimates of previous cohorts and when all studies were stratified by ethnicity, MRI magnet strength, or sequence (Figure 4). However, risk of recurrent ischemic stroke in patients with microbleeds versus no microbleeds was significantly greater in the 2 TIA-only cohorts that had ≤1-year follow-up compared with the other TIA/ischemic stroke cohorts that had >1-year follow-up (relative risk, 4.80; 2.35–9.76 versus 1.62; 1.32–1.99; *P*_het_=0.004; Figure V in the online-only Data Supplement). Sensitivity analysis of all patients revealed broadly similar results (Table VI in the online-only Data Supplement; Figures VI and VII in the online-only Data Supplement).

## Discussion

Our study comprises the 2 largest cohorts to date from the west and the east to report the long-term prognostic implications of microbleed burden in patients with TIA/ischemic stroke, adding ≈6500 patient-years of follow-up data to the 9534 patient-years included in a recent systematic review of 15 smaller studies.^6^ Our study is also the first to report the other nonstroke determinants of the balance of risks and benefits of antiplatelet drugs (extracranial bleeds and coronary events) stratified by microbleed burden, the first to determine the severity and time course of recurrent events, and the first to determine whether risks of microbleeds differ in TIA versus ischemic stroke cohorts.

Our results support those from previous studies that microbleeds represent an imaging biomarker of severe cerebral small vessel disease.^6,27,28^ Similar to previous studies,^1^ we showed that prevalence and burden of microbleeds were significantly greater in Chinese than whites. While this may be because of a greater proportion of hypertension among the Chinese, patients from HKU also consisted entirely of patients with ischemic stroke while ≈53% of OXVASC patients had TIA, in whom the prevalence of microbleeds is known to be lower.^1^ However, when individuals from OXVASC and HKU were stratified by microbleed burden, risk of recurrent stroke was similar regardless of ethnicity, and no heterogeneity was observed when all cohorts were pooled and stratified by ethnicity.

Compared with patients with no microbleeds, TIA/ischemic stroke patients with ≥5 microbleeds on antiplatelet agents were at 3-fold increased risk of recurrent ischemic stroke and at 13-fold increased risk of ICH. However, the relative risk of ischemic versus hemorrhagic events was time dependent, with a 3-fold excess of risk of recurrent ischemic stroke versus ICH in the first year (9.5% versus 3.7%) but an increasing relative risk of ICH thereafter. Furthermore, the disability accrued because of ICH was significantly greater than that because of recurrent ischemic stroke. Our relative risk estimates for ICH in relation to microbleed burden were fairly consistent with the pooled estimates from previous meta-analyses, but our relative risk estimates for recurrent ischemic stroke were more modest.^6^ The pooled estimates from previous meta-analyses were nevertheless undermined by substantial heterogeneity (Figure 4A) because of a 3-fold higher risk of ischemic stroke in TIA-only cohorts compared with cohorts that consisted predominantly of patients with ischemic stroke (Figure V in the online-only Data Supplement). Excluding the TIA-only cohorts^29,30^ substantially reduced the heterogeneity of pooled relative risk estimates of previous studies on recurrent ischemic stroke risk in 1, 2 to 4, and ≥5 microbleeds versus no microbleeds (*P*_het_=0.095 to 0.24; *P*_het_=0.0005 to 0.99; and *P*_het_=0.025 to 0.52, respectively).

Our findings, therefore, have clinical implications on antiplatelet use in TIA/ischemic stroke patients with a high microbleed burden. Taken together with the recent evidence of the considerable early benefit of aspirin in reducing the risk and severity of recurrent ischemic events after TIA/ischemic stroke,^8^ our results suggest that patients with noncardioembolic TIA/ischemic stroke with <5 microbleeds could reasonably be prescribed antiplatelet drugs unless contraindicated or unless future research identifies a clearer hazard. In patients with ≥5 microbleeds however, in view of the high early risk of ischemic events, particularly in patients presenting as a TIA, antiplatelet agents should also be prescribed within the first year of index event. Antiplatelet agents should perhaps be withdrawn thereafter because of the increasing long-term risks of ICH that are more likely to be disabling/fatal than ischemic events.

Although we consider our findings to be valid, our study has limitations. First, several different MRI scanners with different field strengths, echo times, and hemosiderin-sensitive sequences were used for detection of microbleeds. Although all these factors may have affected the sensitivity of microbleed detection,^31–33^ we have shown that scanner strength and sequence did not result in significant heterogeneity of the results (Figure 4). Although there appeared to be a trend toward a greater risk of ICH in TIA/ischemic stroke patients with 1 to 4 microbleeds scanned with T2*-GRE as compared with SWI, studies utilizing SWI are scarce (2 versus 13 studies; 3433 versus 11 234 patient-years), and power to demonstrate heterogeneity between MRIs performed using SWI or T2*-GRE may be limited. Second, the OXVASC and HKU cohorts were different in several aspects—OXVASC was a population-based study, consisting predominantly of whites with 50% being TIAs while the HKU cohort was a hospital-based study comprising predominantly of Chinese with ischemic stroke, which is likely to have accounted for some of the differences in stroke subtypes between the 2 populations. Third, it should be noted that the generalizability of our results are restricted to patients with a probable or definite diagnosis of TIA or ischemic stroke of mild-moderate severity who were fit for a MRI (mean mRS on discharge being 2 for ischemic stroke patients). Also, our results may not be applicable to patients presenting with a transient neurological episode consistent with an amyloid spell and who had clear neuroimaging features suggestive of cerebral amyloid angiopathy (either cortical superficial siderosis or numerous microbleeds). Fourth, we were only able to provide preliminary insights as to whether risks of adverse events among patients with ≥5 microbleeds were time dependent. Although our results were significant, our sample size was small, and confirmation of our results by pooling data from multiple cohorts^34^ would be required before formal clinical recommendations could be made. Fifth, we were not able to determine the prognostic implications of microbleed burden based on location because of small numbers in each subgroup. Previous meta-analysis have nevertheless shown that patients with microbleeds of mixed location are at greatest risk of a recurrent stroke.^6^ Finally, our cohort did not provide data on the prognostic implications of microbleeds among patients on anticoagulants. Several clinical trials are currently underway to answer this question.

In conclusion, in TIA/ischemic stroke patients with ≥5 microbleeds, antiplatelets are likely to be beneficial for secondary prevention of ischemic events within 1 year of index event, especially among those presenting with a TIA where the early ischemic risks are high. However, the associated hemorrhagic risks seem to outweigh its benefits thereafter. Although withholding antiplatelet drugs during the acute phase of TIA/ischemic stroke based on microbleed burden may be inappropriate, the benefits of gradual withdrawal of antiplatelets afterwards needs to be further studied.

## Acknowledgments

We are grateful to all the staff in the general practices that collaborated with the Oxford Vascular Study: Abingdon Surgery, Stert St, Abingdon; Malthouse Surgery, Abingdon; Marcham Road Family Health Centre, Abingdon; The Health Centre, Berinsfield; Key Medical Practice, Kidlington; 19 Beaumont St, Oxford; East Oxford Health Centre, Oxford; Church Street Practice, Wantage. We also acknowledge the use of the facilities of the Acute Vascular Imaging Centre, Oxford, and magnetic resonance imaging unit, Department of Diagnostic Radiology, University of Hong Kong. Dr Lau obtained funding, collected data, did the statistical analysis and interpretation, wrote and revised the article. Dr Lovelock collected data, did the statistical analysis and interpretation, wrote and revised the article. Dr Li acquired data and did the statistical analysis. Drs Simoni and Gutnikov acquired data. Dr Küker provided study supervision and acquired imaging data. Dr Mak provided study supervision and funding, acquired, analyzed, and interpreted imaging data. Dr Rothwell conceived and designed the overall study, provided study supervision and funding, acquired, analyzed, and interpreted data, and wrote and revised the article.

## Sources of Funding

The Oxford Vascular Study is funded by the National Institute for Health Research (NIHR) Oxford Biomedical Research Centre, Wellcome Trust, Wolfson Foundation, British Heart Foundation, and the European Union’s Horizon 2020 programme (grant 666881, SVDs@target). Professor Rothwell is in receipt of a NIHR Senior Investigator award. Magnetic resonance imaging studies from University of Hong Kong (HKU) are funded by the SK Yee Medical Foundation Grant and HKU Strategic Theme in Neurosciences. Dr Lau is funded by a University of Oxford Croucher Scholarship. The views expressed are those of the author(s) and not necessarily those of the National Health Service (NHS), the NIHR, or the Department of Health.

## Disclosures

None.

## Supplementary Material
