# Supplementary material for: Antiplatelet Treatment After Transient Ischemic Attack and Ischemic Stroke in Patients With Cerebral Microbleeds in 2 Large Cohorts and an Updated Systematic Review
Source: Stroke. 2018 May 10;49(6):1434–42. doi: 10.1161/STROKEAHA.117.020104 (PMC5976229; doi:10.1161/STROKEAHA.117.020104)
Supplement: Supplementary file 1 [file str-49-1434-s001.pdf]

SUPPLEMENTAL MATERIAL

Supplementary methods

Perivascular spaces (PVSs) were defined as small (<3mm) punctate (if perpendicular to the plane of scan) or linear (if longitudinal to the plane of scan) hyperintensities on T2 images in the basal ganglia (BG) or centrum semiovale (CS). Burden of PVSs were then stratified into 3 groups: <11, 11-20 and >20. The severity of white matter disease was determined for each patient according to the Fazekas scale. Subcortical white matter hyperintesnity (WMH) was graded as 0 (absent), 1 (puntate foci), 2 (beginning confluence of foci) and 3 (large confluent areas); whilst periventricular WMH was graded as 0 (no WMH except for small triangular foci surrounding the frontal horns), 1 (periventricular hyperintensity surrounding the anterior and posterior horns ± discrete WMHs), 2 (extensive patchy WMHs and their early confluent stages), and 3 (confluent, completely surrounding lateral ventricles). Lacunes were defined as rounded or ovoid lesions, >3 and <20mm in diameter, in the BG, internal capsule, CS or brainstem, of cerebrospinal fluid signal density on T2 and fluid-attenuated inversion recovery (FLAIR) and no increased signal on diffusion weighted imaging (DWI).

Acute coronary syndrome (ACS) was defined as non-ST or ST-segment elevation myocardial infarction based on current guidelines,[1, 2] and sudden cardiac death was defined as a sudden pulseless condition, presumed to be due to a cardiac arrhythmia, in a previously stable individual without a non-cardiac cause of cardiac arrest. Severity of extracranial bleeding were classified according to the CURE trial.[3] Vascular death was defined as death due to lethal cardiac arrhythmias, ACS, congestive heart failure, fatal stroke, pulmonary embolism, aortic dissection or unexplained sudden death.

Supplementary Tables

Supplementary Table I. Imaging sequence parameters of the OXVASC and HKU cohorts

Supplementary Table II. Cause of TIA or ischaemic stroke according to the modified TOAST criteria

Supplementary Table III. Burden of microbleeds in patients with or without anti-thrombotic medications after TIA or ischaemic stroke

Supplementary Table IV. Clinical predictors of a high-burden (≥5) of cerebral microbleeds versus <5 microbleeds

Supplementary Table V. Associations of a high-burden (≥5) of cerebral microbleeds with other neuroimaging markers of small vessel disease versus <5 microbleeds

Supplementary Table VI. Cox regression analyses of risk of adverse events in all TIA / ischaemic stroke patients with increasing burden of microbleeds versus no microbleeds

Supplementary Table VII. Cox regression analyses of risk of adverse events amongst antiplatelet users with increasing burden of microbleeds versus no microbleeds

Supplementary Table VIII. Cox regression analyses of risk of adverse events amongst single antiplatelet users with increasing burden of microbleeds versus no microbleeds

Supplementary Table IX. Summary of studies included in meta-analysis

Supplementary Table I. Imaging sequence parameters of the OXVASC and HKU cohorts

MR parameters	HKU<br>Achieva, Philips Healthcare	OXVASC scanner 1<br>Magnetom Verio, Siemens Healthcare	OXVASC scanner 2<br>Discovery MR750, GE Healthcare	OXVASC scanner 3<br>Achieva, Philips Healthcare	OXVASC scanner 4<br>Signa HDxt, GE Healthcare
**Patients scanned**	1076	388	62	493	137
**Field strength (T)**	3	3	3	1.5	1.5
**T1W TR/TE/TI (ms)**	2000/20/800	2000/1.94/880	-	701/16	-
**T2W TR/TE (ms)**	2377/80	6000/96	5800/94	5061/100	3760/100
**FLAIR TR/TE/TI (ms) (3D)**	4800/282/1650	9000/88/2500	9600/130/2350	11000/140/2800	8080/112/2200
**Diffusion TR/TE (ms)**	2874/46	5300/91	6000/84	2891/73	6100/71
**GRE / SWI TR/TE (ms) (3D)**	SWI 28/23	GRE 504/15	GRE 500/20	GRE 694/23	GRE 560/25
**Pixel bandwidth (Hz)**	218.5 (T1W)<br>350.7 (T2W)<br>144.7 (FLAIR)<br>40.2 (Diffusion)<br>455.7 (SWI)	240 (T1W)<br>220 (T2W)<br>202 (FLAIR)<br>1374 (Diffusion)<br>200 (GRE)	-<br>50 (T2W)<br>41.7 (FLAIR)<br>250 (Diffusion)<br>31.3 (GRE)	87.4 (T1W)<br>88.5 (T2W)<br>375 (FLAIR)<br>25.3 (Diffusion)<br>109.3 (GRE)	-<br>47.6 (T2W)<br>31.3 (FLAIR)<br>-<br>75 (GRE)
**Matrix**	308x207 (T1W)<br>308x235 (T2W)<br>228x227 (FLAIR)<br>112x87 (Diffusion)<br>256x224 (SWI)	256x256 (T1W)<br>320x320 (T2W)<br>192x192 (FLAIR)<br>130x130 (Diffusion)<br>320x256 (GRE)	-<br>512 (T2W)<br>384x224 (FLAIR)<br>128x128 (Diffusion)<br>288x224 (GRE)	118x214 (T1W)<br>356x193 (T2W)<br>236x159 (FLAIR)<br>97x84 (Diffusion)<br>256x163 (GRE)	416x256 (T2W)<br>256x224 (FLAIR)<br>128x128 (Diffusion)<br>288x192 (GRE)
**No. of slices**	25 (T1W)<br>25 (T2W)<br>30 (FLAIR, reconstructed)<br>25 (Diffusion)<br>25 (SWI, reconstructed)	208 (T1W)<br>25 (T2W)<br>50 (FLAIR)<br>25 (Diffusion)<br>25 (GRE)	25	25 (T1W)<br>25 (T2W)<br>28 (FLAIR)<br>25 (Diffusion)<br>22 (GRE)	25
**Slice thickness (mm)**	5 (T1W)<br>5 (T2W)<br>5 (FLAIR coronal), 2.5 (FLAIR axial) (reconstructed)<br>5 (Diffusion)<br>5 (SWI, reconstructed)	1 (T1W)<br>5 (T2W)<br>3 (FLAIR)<br>5 (Diffusion)<br>5 (GRE)	5	5	5
**Inter-slice gap (mm)**	0.5 (T1W)<br>0.5(T2W)<br>0.5 (FLAIR coronal)<br>0 (FLAIR axial)<br>0.5 (Diffusion)<br>0.5 (SWI)	0 (T1W)<br>1 (T2W)<br>0 (FLAIR coronal)<br>1 (Diffusion)<br>1 (GRE)	1	1	1

MR parameters	HKU<br>Achieva, Philips Healthcare	OXVASC scanner 1<br>Magnetom Verio, Siemens Healthcare	OXVASC scanner 2<br>Discovery MR750, GE Healthcare	OXVASC scanner 3<br>Achieva, Philips Healthcare	OXVASC scanner 4<br>Signa HDxt, GE Healthcare
**Voxel size (mm^3)**	0.75x0.95x5.0 (T1W)<br>0.75x0.76x5.0 (T2W)<br>1.10x1.10x0.56 (FLAIR)<br>2.05x2.64x5.0 (Diffusion)<br>0.90x0.90x1.00 (SWI)	1.0x1.0x1.0 (T1W)<br>0.8x0.8x5.0 (T2W)<br>1.0x1.0x3.0 (FLAIR)<br>1.8x1.8x5.0 (Diffusion)<br>0.9x0.8x5.0 (GRE)	-	0.53x0.53x5.0 (T1W)<br>0.65x0.65x5.0 (T2W)<br>0.82x0.81x5.0 (FLAIR)<br>1.74x1.73x5.0 (Diffusion)<br>0.90x0.90x5.0 (GRE)	-

Supplementary Table II. Cause of TIA or ischaemic stroke according to the modified TOAST criteria

	OXVSAC (n=1080)	HKU (n=1003)
**Small vessel occlusion (%)**	132 (12.2)	425 (42.4)
**Large artery atherosclerosis (%)**	145 (13.4)	342 (34.1)
**Cardio-embolism (%)**	167 (15.5)	124 (12.4)
**Multiple causes (%)**	36 (3.3)	28 (2.8)
**Others (%)**	34 (3.2)	18 (1.8)
**Undetermined (%)**	539 (50.0)	44 (4.4)
**Unknown (%)**	26 (2.4)	22 (2.2)

Supplementary Table III. Burden of microbleeds in patients with or without anti-thrombotic medications after TIA or ischaemic stroke

	On anti-thrombotic medications (n=2032)	Not on anti-thrombotic medications (n=50)
**Microbleeds**		
**0**	1442 (71.0)	33 (66.0)
**1-4**	436 (21.5)	11 (22.0)
**5-9**	79 (3.9)	2 (4.0)
**10-24**	49 (2.4)	3 (6.0)
**≥25**	26 (1.3)	1 (2.0)

Supplementary Table IV. Clinical predictors of a high-burden (≥5) of cerebral microbleeds versus <5 microbleeds

	Univariate						Age and sex adjusted		Multi-variate[b] adjusted	
	OXVASC OR (95% CI)	p	HKU OR (95% CI)	p	Combined[a] OR (95% CI)	p	Combined[a] OR (95% CI)	p	Combined[a] OR (95% CI)	p
Age	1.05 (1.02-1.08)	0.001	1.01 (1.00-1.03)	0.096	1.02 (1.01-1.04)	0.001	1.02 (1.01-1.04)	0.001	1.02 (1.00-1.03)	0.035
Male sex	1.00 (0.53-1.89)	1.00	1.26 (0.85-1.88)	0.26	1.18 (0.84-1.65)	0.33	1.27 (0.91-1.79)	0.16	1.25 (0.86-1.80)	0.24
Hypertension	1.14 (0.60-2.15)	0.70	1.65 (1.07-2.55)	0.024	1.47 (1.03-2.10)	0.034	1.32 (0.92-1.90)	0.14	1.38 (0.93-2.03)	0.11
Diabetes	1.41 (0.61-3.25)	0.42	0.88 (0.57-1.36)	0.56	0.96 (0.65-1.42)	0.84	0.92 (0.62-1.35)	0.66	0.83 (0.55-1.26)	0.39
Hyperlipidaemia	1.27 (0.67-2.41)	0.46	0.93 (0.60-1.46)	0.76	1.03 (0.72-1.48)	0.88	1.02 (0.71-1.47)	0.92	0.89 (0.61-1.31)	0.57
Ever-smoker	1.09 (0.58-2.06)	0.78	1.04 (0.68-1.57)	0.87	1.05 (0.74-1.49)	0.77	1.02 (0.71-1.49)	0.90	0.99 (0.68-1.45)	0.96
Atrial fibrillation	1.38 (0.63-3.06)	0.42	0.96 (0.54-1.72)	0.90	1.08 (0.68-1.73)	0.74	0.90 (0.56-1.46)	0.68	0.74 (0.43-1.26)	0.26
Glomerular filtration rate <60ml/min/1.73m^2	1.23 (0.60-2.50)	0.58	1.95 (1.28-2.98)	0.002	1.72 (1.20-2.47)	0.003	1.47 (1.00-2.15)	0.049	1.37 (0.93-2.03)	0.12
Premorbid antiplatelet use	1.76 (0.80-3.91)	0.16	1.38 (0.89-2.14)	0.15	1.46 (0.99-2.14)	0.055	1.28 (0.87-1.90)	0.22	1.42 (0.93-2.15)	0.10
Premorbid anticoagulation use	2.19 (0.28-17.30)	0.46	2.11 (0.77-5.79)	0.15	2.13 (0.86-5.27)	0.10	1.94 (0.78-4.81)	0.16	2.77 (1.00-7.70)	0.050

[a]Adjusted for centre
[b]Adjusted for all variables in univariate analysis
OR, odds ratio; CI, confidence interval

Supplementary Table V. Associations of a high-burden (≥5) of cerebral microbleeds with other neuroimaging markers of small vessel disease versus <5 microbleeds

	Univariate						Age and sex adjusted	
	**OXVASC OR (95% CI)**	**p**	**HKU OR (95% CI)**	**p**	**Combined[a] OR (95% CI)**	**p**	**Combined[a] OR (95% CI)**	**p**
Periventricular white matter hyperintensity	3.25 (2.28-4.65)	<0.0001	3.32 (2.66-4.14)	<0.0001	3.30 (2.73-3.98)	<0.0001	3.45 (2.82-4.21)	<0.0001
Subcortical white matter hyperintensity	3.05 (2.17-4.28)	<0.0001	2.77 (2.18-3.52)	<0.0001	2.86 (2.35-3.48)	<0.0001	2.87 (2.35-3.51)	<0.0001
Lacunes	2.74 (1.39-5.38)	0.003	1.28 (0.87-1.89)	0.21	1.53 (1.08-2.15)	0.016	1.49 (1.06-2.11)	0.023
Basal-ganglia perivascular spaces[b]	2.56 (1.71-3.84)	<0.0001	2.87 (2.18-3.76)	<0.0001	2.77 (2.21-3.48)	<0.0001	2.80 (2.18-3.60)	<0.0001
Centrum-semiovale perivascular spaces[b]	1.88 (1.16-3.06)	0.011	0.76 (0.56-1.03)	0.074	1.01 (0.80-1.29)	0.92	0.96 (0.75-1.22)	0.72

[a]Adjusted for centre
[b]Missing data in 81 patients
OR, odds ratio; CI, confidence interval

Supplementary Table VI. Cox regression analyses of risk of adverse events in all TIA / ischaemic stroke patients with increasing burden of microbleeds versus no microbleeds

	Unadjusted HR (95% CI)*			HR (95% CI)* adjusted for age and sex			HR (95% CI)* adjusted for age, sex and vascular risk factors[a]			
**Microbleed number**	**1**	**2-4**	**≥5**	**1**	**2-4**	**≥5**	**1**	**2-4**	**≥5**	**p_{trend}**
**Recurrent stroke**										
OXVASC	2·06 (1·09-3·89)	2·32 (1·07-5·05)	3·02 (1·45-6·28)	1·75 (0·92-3·34)	2·01 (0·92-4·41)	2·49 (1·18-5·23)	1·70 (0·89-3·24)	1·83 (0·83-4·03)	2·41 (1·14-5·10)	0·0005
HKU	1·32 (0·79-2·21)	1·59 (0·93-2·70)	2·86 (1·77-4·61)	1·25 (0·74-2·09)	1·38 (0·81-2·36)	2·54 (1·57-4·11)	1·30 (0·78-2·19)	1·36 (0·79-2·33)	2·81 (1·73-4·57)	0·0002
Combined[b]	1.56 (1.04-2.33)	1.82 (1.17-2.83)	3.03 (2.04-4.51)	1.40 (0.93-2.09)	1.56 (1.00-2.43)	2.59 (1.74-3.86)	1.38 (0.93-2.07)	1.46 (0.94-2.28)	2.67 (1.80-3.98)	<0·0001
**Ischaemic stroke**										
OXVASC	1·98 (1·02-3·86)	1·34 (0·49-3·68)	2·84 (1·30-6·19)	1·72 (0·88-3·39)	1·16 (0·42-3·21)	2·42 (1·09-5·35)	1·65 (0·84-3·24)	0·98 (0·35-2·74)	2·32 (1·04-5·16)	0·060
HKU	1·41 (0·83-2·41)	1·49 (0·84-2·65)	1·78 (0·99-3·21)	1·33 (0·78-2·27)	1·29 (0·72-2·30)	1·58 (0·88-2·86)	1·40 (0·82-2·39)	1·28 (0·71-2·30)	1·83 (1·01-3·33)	0·054
Combined[b]	1.61 (1.06-2.44)	1.52 (0.93-2.49)	2.10 (1.31-3.38)	1.44 (0.95-2.19)	1.30 (0.79-2.14)	1.81 (1.13-2.91)	1.42 (0.94-2.16)	1.19 (0.73-1.96)	1.87 (1.16-3.01)	0.015
**Intracerebral haemorrhage**										
OXVASC	2·69 (0·31-23·06)	14·37 (3·42-60·34)	4·94 (0·58-42·41)	1·96 (0·22-17·11)	14·40 (3·28-63·11)	3·09 (0·35-27·16)	1·95 (0·21-18·04)	15·43 (3·21-74·31)	3·80 (0·41-35·33)	0·011
HKU	0·58 (0·07-4·92)	2·33 (0·56-9·73)	11·13 (3·86-32·04)	0·54 (0·06-4·65)	2·08 (0·49-8·78)	10·12 (3·49-29·35)	0·59 (0·07-5·05)	2·14 (0·50-9·12)	9·51 (3·25-27·81)	<0·0001
Combined[b]	1.05 (0.22-4.93)	4.53 (1.57-13.10)	11.35 (4.66-27.65)	0.93 (0.20-4.34)	3.93 (1.35-11.42)	9.52 (3.89-23.30)	0.91 (0.20-4.26)	4.09 (1.40-11.95)	9.81 (3.98-24.15)	<0·0001

	Unadjusted HR (95% CI)*			HR (95% CI)* adjusted for age and sex			HR (95% CI)* adjusted for age, sex and vascular risk factors[a]			
**Microbleed number**	**1**	**2-4**	**≥5**	**1**	**2-4**	**≥5**	**1**	**2-4**	**≥5**	**p_{trend}**
**Acute coronary event**										
OXVASC	1·27 (0·30-5·43)	-	3·64 (1·08-12·26)	0·82 (0·19-3·52)	-	2·03 (0·59-6·94)	0·90 (0·21-3·87)	-	1·96 (0·57-6·81)	0·75
HKU	0·80 (0·36-1·74)	1·11 (0·51-2·43)	1·19 (0·52-2·71)	0·73 (0·33-1·60)	0·89 (0·40-1·95)	0·95 (0·42-2·18)	0·75 (0·34-1·64)	0·88 (0·40-1·95)	1·01 (0·44-2·33)	0·86
Combined[b]	0.88 (0.44-1.77)	0.99 (0.46-2.13)	1.53 (0.76-3.08)	0.73 (0.37-1.47)	0.76 (0.36-1.63)	1.15 (0.58-2.30)	0.73 (0.36-1.46)	0.72 (0.33-1.55)	1.23 (0.61-2.47)	0.98
**Ischaemic stroke and acute coronary event**										
OXVASC	1·72 (0·92-3·23)	1·05 (0·39-2·86)	3·17 (1·65-6·11)	1·39 (0·74-2·62)	0·83 (0·30-2·26)	2·48 (1·27-4·84)	1·35 (0·72-2·55)	0·74 (0·27-2·05)	2·33 (1·19-4·56)	0·066
HKU	1·15 (0·74-1·80)	1·32 (0·82-2·11)	1·39 (0·84-2·31)	1·08 (0·69-1·68)	1·11 (0·69-1·78)	1·20 (0·72-2·00)	1·11 (0·71-1·74)	1·10 (0·68-1·77)	1·37 (0·82-2·28)	0·27
Combined[b]	1.33 (0.92-1.92)	1.33 (0.88-2.03)	1.80 (1.20-2.73)	1.16 (0.81-1.68)	1.09 (0.72-1.67)	1.50 (0.99-2.25)	1.15 (0.80-1.66)	1.02 (0.67-1.55)	1.56 (1.04-2.34)	0.085
**Major extracranial bleeding[c]**										
OXVASC	0·77 (0·10-5·82)	1·34 (0·18-10·10)	1·47 (0·20-11·07)	0·53 (0·07-4·06)	1·05 (0·14-8·05)	0·96 (0·13-7·33)	0·47 (0·06-3·64)	1·02 (0·13-7·90)	1·00 (0·13-7·73)	0·85
HKU	0·49 (0·11-2·17)	0·64 (0·14-2·88)	2·03 (0·72-5·77)	0·46 (0·10-2·05)	0·56 (0·13-2·53)	1·81 (0·63-5·15)	0·42 (0·09-1·91)	0·70 (0·15-3·18)	2·19 (0·75-6·38)	0·36
Combined[b]	0.57 (0.17-1.90)	0.79 (0.23-2.67)	1.93 (0.77-4.80)	0.49 (0.15-1.64)	0.67 (0.20-2.25)	1.57 (0.63-3.91)	0.47 (0.14-1.58)	0.71 (0.21-2.40)	1.71 (0.69-4.26)	0.59

	Unadjusted HR (95% CI)*			HR (95% CI)* adjusted for age and sex			HR (95% CI)* adjusted for age, sex and vascular risk factors[a]			
**Microbleed number**	**1**	**2-4**	**≥5**	**1**	**2-4**	**≥5**	**1**	**2-4**	**≥5**	p_{trend}
**All-cause mortality**										
OXVASC	2·22 (1·37-3·61)	2·20 (1·22-4·00)	2·21 (1·16-4·21)	1·44 (0·88-2·35)	1·62 (0·89-2·95)	1·24 (0·65-2·38)	1·30 (0·79-2·14)	1·41 (0·76-2·62)	1·26 (0·65-2·43)	0·20
HKU	0·97 (0·59-1·59)	1·48 (0·92-2·39)	1·81 (1·12-2·92)	0·86 (0·52-1·41)	1·16 (0·72-1·88)	1·48 (0·92-2·39)	0·89 (0·54-1·46)	1·19 (0·73-1·93)	1·54 (0·95-2·49)	0·094
Combined[b]	1.43 (1.00-2.04)	1.80 (1.24-2.63)	2.07 (1.41-3.04)	1.10 (0.77-1.56)	1.33 (0.92-1.94)	1.43 (0.98-2.09)	1.05 (0.74-1.50)	1.24 (0.85-1.81)	1.46 (1.00-2.14)	0.041
**Vascular death**										
OXVASC	1·54 (0·55-4·35)	-	1·46 (0·35-6.09)	1·01 (0·36-2·87)	-	0·84 (0·20-3·52)	0·98 (0·34-2·80)	-	0·81 (0·19-3·41)	0·34
HKU	1·15 (0·57-2·31)	1·74 (0·88-3·42)	1·59 (0·75-3·37)	1·04 (0·52-2·10)	1·39 (0·70-2·73)	1·27 (0·60-2·70)	1·09 (0·54-2·20)	1·37 (0·69-2·74)	1·38 (0·65-2·95)	0·29
Combined[b]	1.21 (0.68-2.17)	1.38 (0.73-2.61)	1.53 (0.79-2.96)	1.00 (0.56-1.79)	1.07 (0.57-2.03)	1.11 (0.58-2.14)	1.00 (0.56-1.78)	1.02 (0.54-1.93)	1.17 (0.61-2.25)	0.71
**Non-vascular death**										
OXVASC	2·42 (1·34-4·36)	3·17 (1·68-5.99)	2·10 (0·91-4·82)	1·57 (0·87-2·85)	2·30 (1·21-4·37)	1·20 (0·52-2·78)	1·41 (0·77-2·59)	2·08 (1.07-4·05)	1·28 (0·55-2·99)	0·084
HKU	0·81 (0·40-1·63)	1·26 (0·64-2·48)	1·95 (1·05-3·62)	0·68 (0·34-1·39)	0·97 (0·49-1·91)	1·59 (0·86-2·97)	0·70 (0·34-1·42)	1·04 (0·52-2·07)	1·57 (0·84-2·93)	0·24
Combined[b]	1.48 (0.93-2.35)	2.05 (1.27-3.29)	2.27 (1.39-3.72)	1.11 (0.70-1.76)	1.48 (0.92-2.38)	1.56 (0.96-2.54)	1.06 (0.67-1.68)	1.41 (0.87-2.28)	1.60 (0.98-2.61)	0.037

*0 microbleeds as reference
[a]Hypertension, hyperlipidemia, diabetes, atrial fibrillation, smoking
[b]Also adjusted for centre
[c]Dental and nasal bleeds excluded
HR, hazards ratio; CI, confidence interval

Supplementary Table VII. Cox regression analyses of risk of adverse events amongst antiplatelet users with increasing burden of microbleeds versus no microbleeds

	Unadjusted HR (95% CI)*			HR (95% CI)* adjusted for age and sex			HR (95% CI)* adjusted for age, sex and vascular risk factors[a]			
**Microbleed number**	**1**	**2-4**	**≥5**	**1**	**2-4**	**≥5**	**1**	**2-4**	**≥5**	p_{trend}
**Recurrent stroke**										
OXVASC	2·00 (0·99-4·03)	2·63 (1·14-6·08)	3·47 (1·66-7·24)	1·72 (0·85-3·50)	2·27 (0·97-5·30)	2·92 (1·38-6·17)	1·55 (0·76-3·15)	1·83 (0·78-4·31)	2·81 (1·31-6·00)	0·003
HKU	1·38 (0·79-2·41)	1·44 (0·78-2·66)	3·07 (1·83-5·15)	1·27 (0·72-2·22)	1·27 (0·69-2·36)	2·71 (1·61-4·56)	1·30 (0·74-2·29)	1·28 (0·69-2·38)	2·89 (1·70-4·90)	0·001
Combined[b]	1.61 (1.03-2.50)	1.77 (1.07-2.92)	3.35 (2.20-5.10)	1.43 (0.92-2.22)	1.53 (0.93-2.53)	2.87 (1.88-4.39)	1.38 (0.89-2.13)	1.39 (0.84-2.30)	2.89 (1.89-4.41)	<0·0001
**Ischaemic stroke**										
OXVASC	2·13 (1·06-4·31)	1·76 (0·64-4·84)	3·24 (1·48-7·09)	1·88 (0·92-3·83)	1·51 (0·54-4·21)	2·80 (1·26-6·21)	1·66 (0·81-3·40)	1·13 (0·40-3·21)	2·65 (1·18-5·95)	0·025
HKU	1·41 (0·79-2·52)	1·34 (0·69-2·57)	1·81 (0·96-3·43)	1·30 (0·73-2·33)	1·18 (0·61-2·27)	1·60 (0·84-3·03)	1·35 (0·75-2·42)	1·21 (0·62-2·36)	1·81 (0·95-3·45)	0·096
Combined[b]	1.69 (1.08-2.66)	1.53 (0.88-2.65)	2.29 (1.39-3.79)	1.51 (0.96-2.38)	1.32 (0.76-2.30)	1.98 (1.20-3.28)	1.44 (0.92-2.26)	1.16 (0.67-2.02)	2.01 (1.22-3.32)	0.013
**Intracerebral haemorrhage**										
OXVASC	-	12·90 (2·36-70·47)	6·35 (0·71-56·99)	-	12·74 (2·22-72·97)	4·20 (0·46-38·62)	-	10·02 (1·67-60·18)	4·51 (0·43-47·36)	0·042
HKU	0·95 (0·10-9·12)	2·57 (0·43-15·40)	16·62 (4·57-60·41)	0·86 (0·09-8·32)	2·30 (0·38-13·85)	14·91 (4·07-54·65)	0·88 (0·09-8·55)	2·22 (0·37-13·42)	13·15 (3·54-48·93)	<0·0001
Combined[b]	0.73 (0.09-6.05)	4.21 (1.16-15.27)	14.34 (5.22-39.39)	0.63 (0.08-5.24)	3.67 (1.01-13.33)	11.86 (4.29-32.81)	0.62 (0.07-5.13)	3.81 (1.04-13.96)	11.52 (4.09-32.43)	<0·0001
**Acute coronary event**										
OXVASC	1·69 (0·39-7·34)	-	3·01 (0·69-13·10)	1·14 (0·26-4·99)	-	1·76 (0·40-7·76)	1·18 (0·27-5·17)	-	1·62 (0·36-7·34)	0·95
HKU	1·15 (0·51-2·61)	1·03 (0·39-2·74)	1·47 (0·59-3·66)	1·02 (0·45-2·33)	0·85 (0·32-2·28)	1·16 (0·46-2·89)	1·06 (0·46-2·42)	0·92 (0·34-2·48)	1·08 (0·42-2·76)	0·95
Combined[b]	1.25 (0.61-2.58)	0.89 (0.34-2.32)	1.72 (0.78-3.78)	1.01 (0.49-2.07)	0.71 (0.27-1.83)	1.28 (0.58-2.80)	0.99 (0.48-2.04)	0.67 (0.26-1.74)	1.34 (0.61-2.95)	0.85

	**Unadjusted HR (95% CI)***			**HR (95% CI)* adjusted for age and sex**			**HR (95% CI)* adjusted for age, sex and vascular risk factors[a]**			
**Microbleed number**	**1**	**2-4**	**≥5**	**1**	**2-4**	**≥5**	**1**	**2-4**	**≥5**	**p_{trend}**
**Ischaemic stroke and acute coronary event**										
OXVASC	1·92 (0·99-3·73)	1·40 (0·51-3·83)	3·33 (1·66-6·65)	1·59 (0·82-3·11)	1·09 (0·40-3·00)	2·68 (1·33-5·42)	1·43 (0·73-2·80)	0·88 (0·31-2·45)	2·46 (1·21-5·03)	0·041
HKU	1·29 (0·80-2·09)	1·25 (0·73-2·16)	1·48 (0·84-2·58)	1·18 (0·73-1·91)	1·08 (0·62-1·86)	1·27 (0·72-2·22)	1·22 (0·75-1·98)	1·10 (0·63-1·92)	1·40 (0·80-2·48)	0·28
Combined[b]	1.52 (1.02-2.25)	1.36 (0.84-2.20)	1.97 (1.26-3.07)	1.32 (0.89-1.95)	1.13 (0.70-1.82)	1.63 (1.05-2.54)	1.28 (0.86-1.89)	1.02 (0.63-1.64)	1.68 (1.08-2.61)	0.058
**Major extracranial bleed[c]**										
OXVASC	1·25 (0·16-9·81)	-	-	0·92 (0·12-7·31)	-	-	0·79 (0·10-6·52)	-	-	0·35
HKU	0·71 (0·15-3·36)	-	2·43 (0·73-8·07)	0·62 (0·13-2·92)	-	2·08 (0·62-6·94)	0·66 (0·14-3·22)	-	2·32 (0·64-8·40)	0·53
Combined[b]	0.81 (0.23-2.85)	-	1.85 (0.60-5.66)	0.68 (0.20-2.39)	-	1.50 (0.49-4.60)	0.66 (0.19-2.31)	-	1.53 (0.49-4.75)	0.87
**All-cause mortality**										
OXVASC	3·00 (1·83-4·91)	2·16 (1·09-4·29)	2·42 (1·22-4·80)	1·97 (1·20-3·23)	1·55 (0·78-3·09)	1·42 (0·71-2·83)	1·66 (1·00-2·76)	1·22 (0·60-2·51)	1·54 (0·77-3·08)	0·10
HKU	1·08 (0·61-1·91)	1·73 (1·00-3·00)	2·18 (1·27-3·74)	0·88 (0·49-1·57)	1·42 (0·82-2·47)	1·75 (1·02-3·01)	0·90 (0·51-1·61)	1·56 (0·89-2·72)	1·63 (0·93-2·86)	0·046
Combined[b]	1.80 (1.22-2.66)	2.02 (1.31-3.10)	2.46 (1.61-3.74)	1.33 (0.90-1.95)	1.50 (0.98-2.30)	1.66 (1.09-2.51)	1.23 (0.84-1.80)	1.36 (0.88-2.09)	1.66 (1.09-2.54)	0.012
**Vascular death**										
OXVASC	2·41 (0·83-6.98)	-	2·16 (0·51-9.17)	1·62 (0·56-4·71)	-	1·38 (0·32-5·89)	1·49 (0·50-4·40)	-	1·35 (0·31-5·85)	0·97
HKU	1·37 (0·59-3·18)	2·46 (1·12-5·37)	2·04 (0·84-4·91)	1·20 (0·52-2·80)	2·06 (0·94-4·51)	1·59 (0·66-3·85)	1·24 (0·53-2·89)	2·17 (0·98-4·81)	1·55 (0·63-3·84)	0·12
Combined[b]	1.59 (0.81-3.10)	1.92 (0.94-3.95)	2.03 (0.96-4.28)	1.29 (0.66-2.51)	1.56 (0.76-3.19)	1.45 (0.69-3.05)	1.27 (0.65-2.46)	1.50 (0.73-3.08)	1.48 (0.70-3.14)	0.18

	Unadjusted HR (95% CI)*			HR (95% CI)* adjusted for age and sex			HR (95% CI)* adjusted for age, sex and vascular risk factors[a]			
**Microbleed number**	**1**	**2-4**	**≥5**	**1**	**2-4**	**≥5**	**1**	**2-4**	**≥5**	p_{trend}
**Non-vascular death**										
OXVASC	3·10 (1·70-5·65)	3·26 (1·61-6.59)	2.02 (0·81-5.03)	2·05 (1·12-3·75)	2·27 (1·11-4·62)	1·19 (0·48-2.99)	1·74 (0·94-3·23)	1.84 (0·88-3.89)	1·36 (0·54-3·44)	0·089
HKU	0·87 (0·39-1·92)	1·25 (0·57-2·77)	2·26 (1·14-4·48)	0·65 (0·29-1·45)	1·00 (0·45-2·22)	1·87 (0·94-3·70)	0·66 (0·30-1·48)	1·10 (0·49-2·46)	1·59 (0·76-3·31)	0·27
Combined[b]	1.81 (1.10-2.97)	2.13 (1.24-3.65)	2.50 (1.46-4.27)	1.30 (0.79-2.12)	1.52 (0.89-2.60)	1.66 (0.98-2.83)	1.18 (0.72-1.94)	1.36 (0.79-2.35)	1.68 (0.98-2.89)	0.044

[*]0 microbleeds as reference
[a]Hypertension, hyperlipidemia, diabetes, atrial fibrillation, smoking
[b]Also adjusted for centre
[c]Dental and nasal bleeds excluded
HR, hazards ratio; CI, confidence interval

Supplementary Table VIII. Cox regression analyses of risk of adverse events amongst single antiplatelet users with increasing burden of microbleeds versus no microbleeds

	Unadjusted HR (95% CI)*			HR (95% CI)* adjusted for age and sex			HR (95% CI)* adjusted for age, sex and vascular risk factors[a]			
**Microbleed number**	**1**	**2-4**	**≥5**	**1**	**2-4**	**≥5**	**1**	**2-4**	**≥5**	p_{trend}
**Recurrent stroke**	1.83 (1.11-3.02)	1.92 (1.09-3.38)	4.17 (2.63-6.63)	1.62 (0.98-2.66)	1.64 (0.93-2.90)	3.53 (2.22-5.62)	1.52 (0.93-2.49)	1.52 (0.86-2.68)	3.46 (2.17-5.51)	<0·0001
**Ischaemic stroke**	1.89 (1.14-3.16)	1.96 (1.09-3.52)	2.82 (1.63-4.86)	1.66 (1.00-2.76)	1.67 (0.93-3.00)	2.37 (1.38-4.10)	1.55 (0.93-2.57)	1.51 (0.84-2.72)	2.36 (1.37-4.08)	0.002
**Intracerebral haemorrhage**	1.34 (0.14-13.14)	1.78 (0.18-17.68)	22.19 (5.84-84.28)	1.25 (0.13-12.33)	1.64 (0.16-16.40)	19.89 (5.15-76.82)	1.33 (0.13-13.25)	1.88 (0.19-19.08)	19.53 (4.83-79.02)	<0·0001
**Acute coronary event**	1.48 (0.68-3.21)	0.45 (0.11-1.91)	1.88 (0.81-4.41)	1.18 (0.55-2.55)	0.36 (0.09-1.54)	1.39 (0.60-3.23)	1.15 (0.53-2.49)	0.34 (0.08-1.45)	1.40 (0.59-3.30)	0.99
**Ischaemic stroke and acute coronary event**	1.76 (1.14-2.72)	1.48 (0.87-2.53)	2.32 (1.43-3.75)	1.51 (0.98-2.33)	1.22 (0.71-2.08)	1.89 (1.17-3.07)	1.45 (0.94-2.22)	1.12 (0.65-1.92)	1.93 (1.19-3.12)	0.017
**Major extracranial bleeding[b]**	0.65 (0.14-2.91)	-	2.11 (0.67-6.64)	0.55 (0.12-2.46)	-	1.73 (0.55-5.44)	0.52 (0.12-2.36)	-	1.64 (0.50-5.36)	0.95
**All-cause mortality**	2.10 (1.37-3.22)	2.05 (1.24-3.39)	2.84 (1.78-4.52)	1.52 (0.99-2.32)	1.55 (0.94-2.55)	1.90 (1.20-3.02)	1.40 (0.91-2.14)	1.56 (0.95-2.59)	1.98 (1.24-3.18)	0.002
**Vascular death**	2.24 (1.10-4.53)	1.81 (0.77-4.27)	2.44 (1.08-5.51)	1.80 (0.89-3.63)	1.48 (0.63-3.47)	1.73 (0.77-3.89)	1.83 (0.90-3.69)	1.54 (0.65-3.65)	1.87 (0.82-4.25)	0.094
**Non-vascular death**	1.77 (1.00-3.12)	2.18 (1.17-4.06)	2.65 (1.45-4.82)	1.25 (0.71-2.20)	1.60 (0.86-2.97)	1.74 (0.96-3.16)	1.11 (0.63-1.95)	1.58 (0.85-2.94)	1.81 (0.98-3.31)	0.033

[*]0 microbleeds as reference, adjusted for centre
[a]Hypertension, hyperlipidemia, diabetes, atrial fibrillation, smoking
[b]Dental and nasal bleeds excluded
HR, hazards ratio; CI, confidence interval

Supplementary Table IX. Summary of studies included in meta-analysis

Author	Region	Year	N and disease type (% TIA)	MRI	Mean age, yr	Males (%)	Antiplatelets (%)	Anticoagulants (%)	Follow-up (patient yrs)
**Western cohorts**									
Lau, Lovelock *et al*.	UK	2017	1080 IS/TIA (53)	1·5/3T T2* GRE	68	53	88	11	4265
CROMIS I[a]	UK	NA	68 IS/TIA	1·5T T2*GRE	66	66	81	16	136
Heidelberg[a]	NA	NA	265 IS	SWI	65	67	78	20	265
Kwa *et al*.[4]	Netherlands	2013	397 IS/TIA (49)	0·5/1/1·5T T2* GRE	65	58	90	10	1522
Fluri *et al*.[5]	Switzerland	2011	176 TIA	T2* GRE	69	61	77	12	44
Thijs *et al*.[6]	Belgium	2010	487 IS/TIA (27)	1/1·5/3T T2* GRE	72	61	73	27	812
Boulanger *et al*.[7]	Canada	2006	236 IS/TIA	3T T2* GRE	NA	55	NA	NA	354
**Asian cohorts**									
Lau *et al*.[8]	Hong Kong, China	2017	1003 IS	3T SWI	69	60	88	10	3168
Lim, *et al*.[9]	Korea	2015	500 TIA	NA T2* GRE	64	58	91	15	125
Song, *et al*.[10]	Korea	2013	550 IS	3T MRI	70	59	35	87	1375
Mok et al.[11]	Hong Kong, China	2009	75 IS	1.5T T2* GRE	71	52	96	0	375
Soo *et al*.[12]	Hong Kong, China	2008	908 IS	1·5T T2* GRE	68	58	93	3	2059
Huang *et al*.[13]	China	2008	636 IS	1·5T T2* GRE	60	69	100	0	742
Naka *et al*.[14]	Japan	2006	183 Lacunar and atherothrombotic stroke	1T T2* GRE	NA	NA	93	2	275
Fan *et al*.[15]	China	2003	121 IS	1.5T T2* GRE	68	68	80	6	272
Imaizumi *et al*.[16]	Japan	NA	138 IS	1·5T T2* GRE	66	66	33	2	253

[a]Unpublished cohorts
IS, ischaemic stroke; TIA, transient ischaemic attack; GRE, gradient echo; SWI, susceptibility weighted imaging; NA, not available

Supplementary Figures

Supplementary Figure I. Flow chart of literature search and additional study selection since most recent meta-analysis

Supplementary Figure II. Severity of recurrent stroke (mRS at 1-month) in all antiplatelet users (A) and in antiplatelet users with ≥5 microbleeds (B)

Supplementary Figure III. Pooled analyses of relative risk estimates from the current and previous studies showing risk of recurrent ischaemic stroke amongst TIA / ischaemic stroke patients on antiplatelet agents with microbleeds versus those without

Supplementary Figure IV. Pooled analyses of relative risk estimates from the current and previous studies showing risk of intracerebral haemorrhage amongst TIA / ischaemic stroke patients on antiplatelet agents with microbleeds versus those without

Supplementary Figure V. Pooled analyses of relative risk estimates from the current and previous studies showing risk of recurrent ischaemic stroke (A) and intracerebral hemorrhage (B) in TIA/ischaemic stroke patinets on antiplatelet agents, stratified by presence versus absence of microbleeds

Supplementary Figure VI. Pooled analyses of relative risk estimates from the current and previous studies showing risk of recurrent ischaemic stroke amongst TIA / ischaemic stroke patients with microbleeds versus those without

Supplementary Figure VII. Pooled analyses of relative risk estimates from the current and previous studies showing risk of intracerebral haemorrhage amongst TIA / ischaemic stroke patients with microbleeds versus those without

Supplemenary Figure I. Flow chart of literature search and additional study selection since most recent meta-analysis

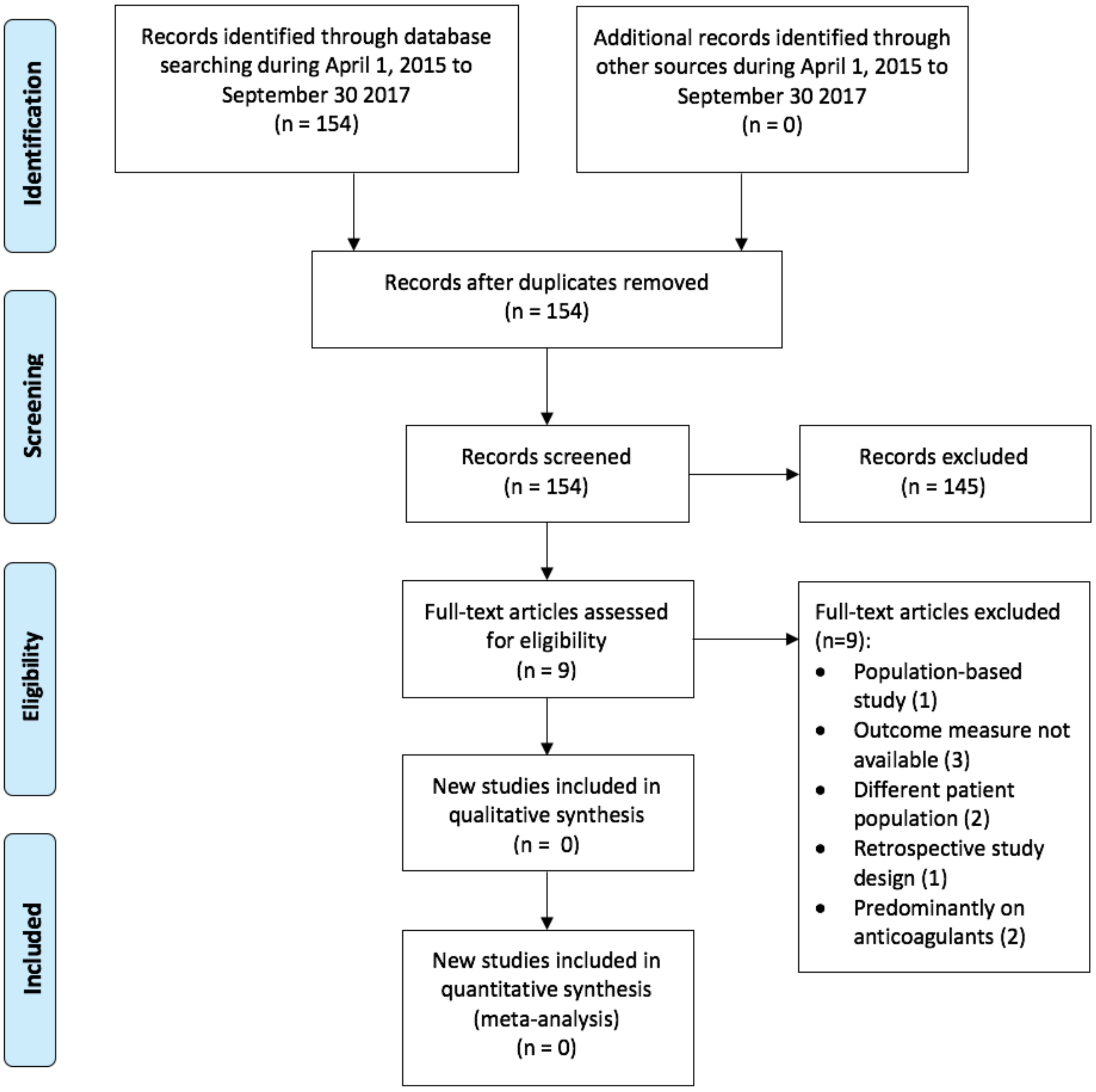

Supplementary Figure II. Severity of recurrent stroke (mRS at 1-month) in all antiplatelet users (A) and in antiplatelet users with ≥5 microbleeds (B)

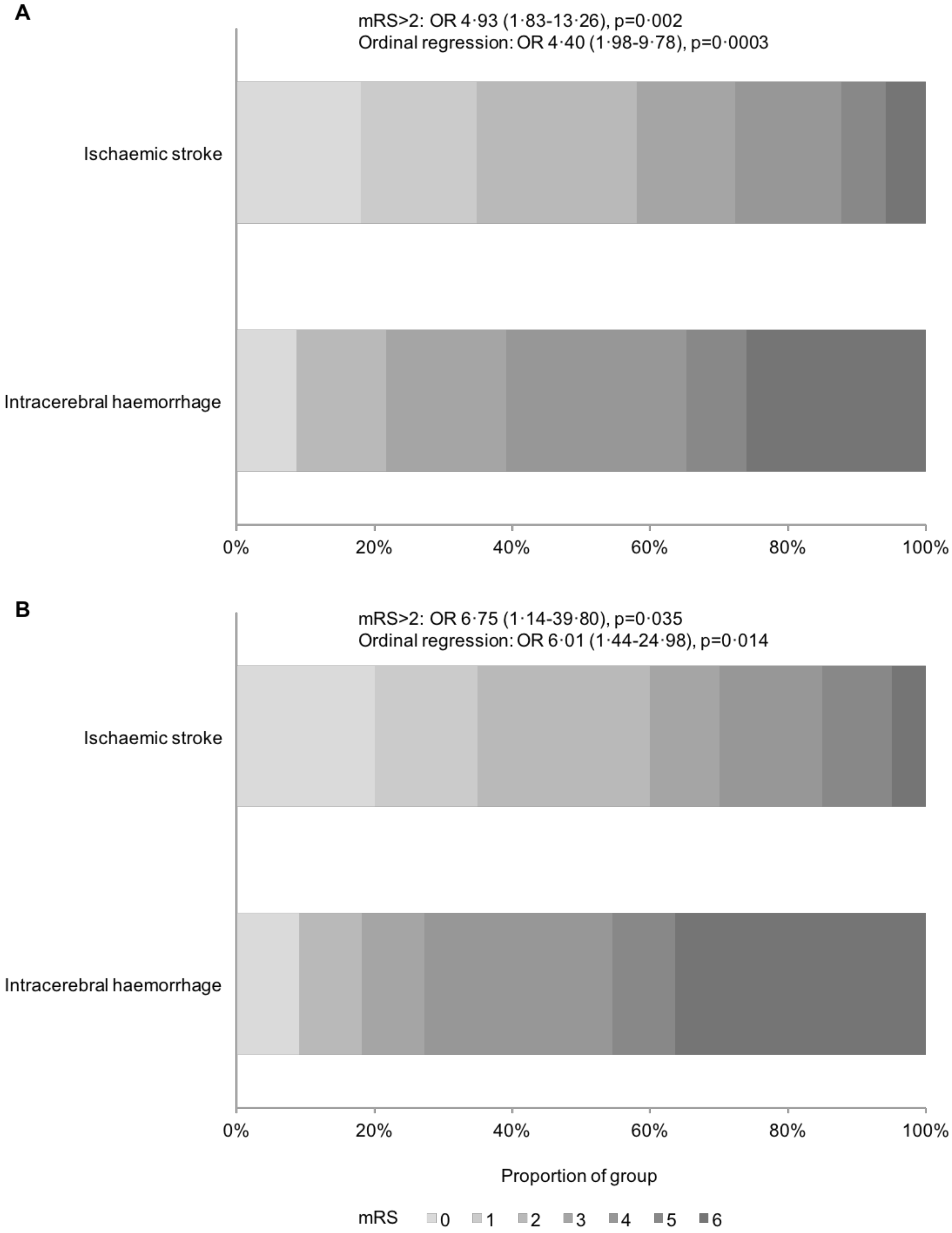

OR=odds ratio; mRS=modified Rankin Scale

Supplementary Figure III. Pooled analyses of relative risk estimates from the current and previous studies showing risk of recurrent ischaemic stroke amongst TIA / ischaemic stroke patients on antiplatelet agents with microbleeds versus those without

1 vs. no microbleeds

Study	RR	95% CI
Huang	1·46	0·32-6·73
Naka	2·18	0·33-14·39
Soo	0·57	0·25-1·28
Fluri	8·82	1·93-40·33
Thijs	1·40	0·49-3·95
Heidelberg	1·60	0·20-12·68
Kwa	2·12	0·66-6·83
Lim	3·09	0·22-42·97
CROMIS I	3·40	1·17-9·89
**Prevoius studies**	**1·86**	**1·03-3·34**
**Significance p=0·040, heterogeneity p=0·095**		
**OXVASC and HKU**	**1·56**	**1·03-2·38**
**Significance p=0·038, heterogeneity p=0·54**		
**Overall**	**1·68**	**1·14-2·48**
**Significance p=0·009, heterogeneity p=0·18**		

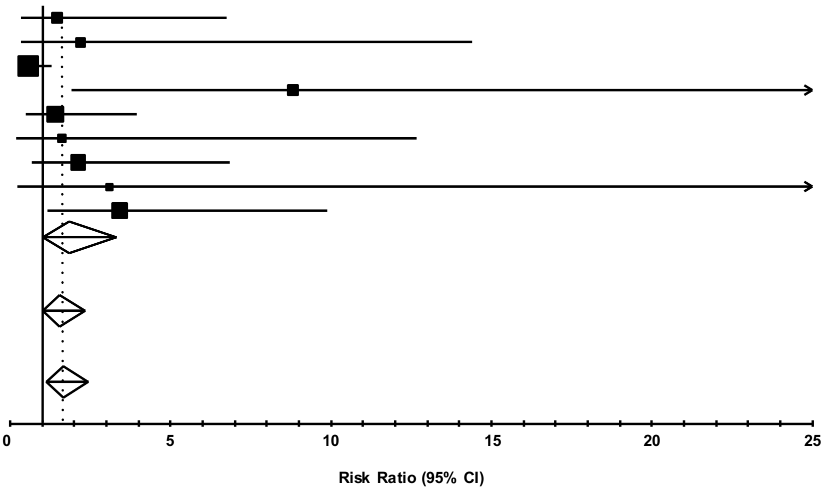

2-4 vs. no microbleeds

Study	RR	95% CI
Huang	2·25	0·75-6·75
Naka	1·79	0·53-5·98
Soo	2·21	1·31-3·71
Fluri	40·06	13·94-115·13
Thijs	1·65	0·64-4·25
Heidelberg	1·09	0·06-18·39
Kwa	3·16	0·81-12·26
Lim	2·03	0·50-8·27
CROMIS 1	1·70	0·25-11·45
**Previous studies**	**2·93**	**1·42-6·04**
**Significance p=0·004, heterogeneity p=0·0005**		
**OXVASC and HKU**	**1·41**	**0·84-2·37**
**Significance p=0·20, heterogeneity p=0·64**		
**OVERALL**	**2·51**	**1·41-4·47**
**Significance p=0·002, heterogeneity p=0·0003**		

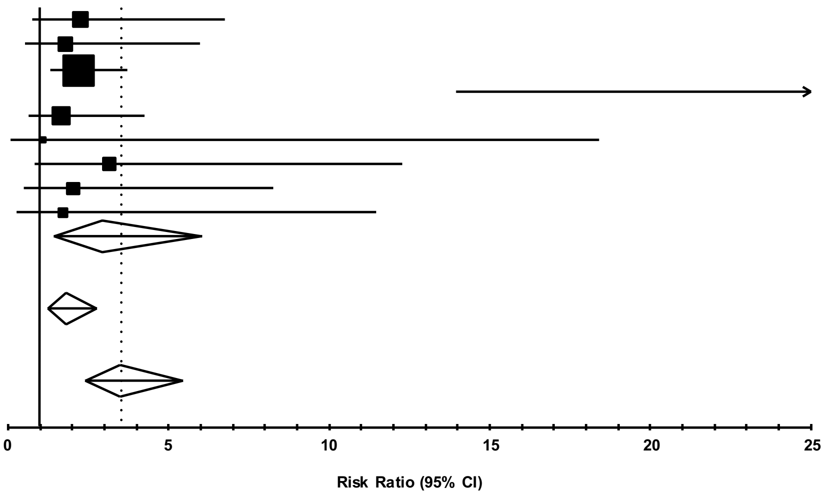

≥5 vs. no microbleeds

Study	RR	95% CI
Huang	3·76	1·34-10·53
Naka	0·25	0·02-4·15
Soo	1·56	0·88-2·75
Fluri	16·67	2·36-117·55
Thijs	2·69	0·98-7·37
Heidelberg	1·95	0·25-15·35
Kwa	3·42	0·54-21·72
Lim	8·46	3·63-19·74
CROMIS 1	2·13	0·33-13·60
**Previous studies**	**3·08**	**1·65-5·76**
**Significance p=0·0004, heterogeneity p=0·025**		
**OXVASC and HKU**	**2·08**	**1·25-3·46**
**Significance p=0·005, heterogeneity p=0·27**		
**OVERALL**	**2·75**	**1·75-4·34**
**Significance p<0·0001, heterogeneity p=0·031**		

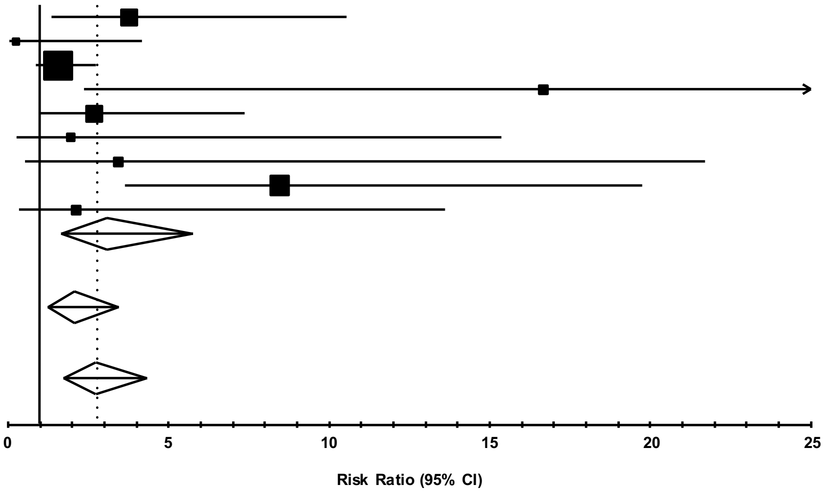

Supplementary Figure IV. Pooled analyses of relative risk estimates from the current and previous studies showing risk of intracerebral haemorrhage amongst TIA / ischaemic stroke patients on antiplatelet agents with microbleeds versus those without

1 vs. no microbleeds

Study	RR	95% CI
Huang	17·33	0·71-420·91
Naka	21·83	1·55-308·50
Soo	3·04	0·56-16·38
Thijs	2·18	0·09-52·74
Kwa	1·30	0·07-23·51
**Previous studies**	**4·51**	**1·48-13·72**
**Significance: p=0·008, heterogeneity p=0·53**		
**OXVASC and HKU**	**0·84**	**0·10-7·02**
**Significance p=0·86, heterogeneity p=0·73**		
**OVERALL**	**3·14**	**1·17-8·42**
**Significance p=0·023, heterogeneity p=0·52**		

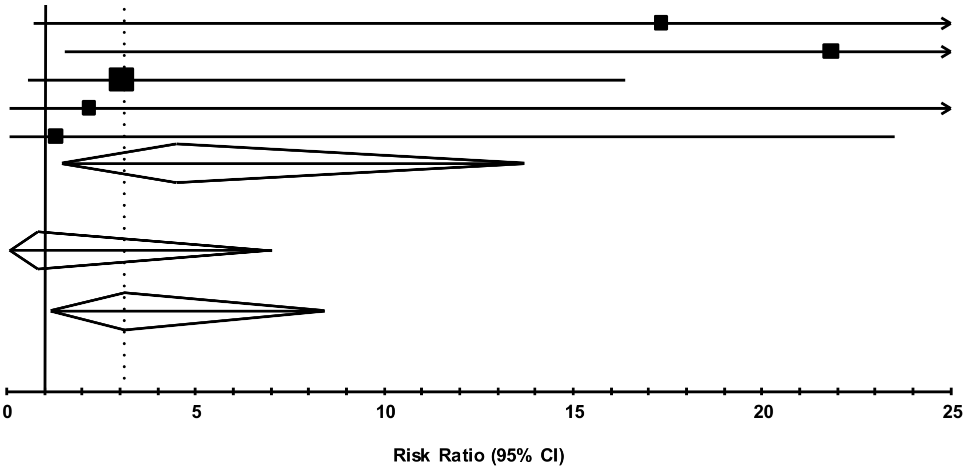

2-4 vs. no microbleeds

Study	RR	95% CI
Huang	10·75	0·44-262·01
Naka	1·91	0·08-45·54
Soo	7·57	1·73-33·09
Thijs	6·28	0·40-99·00
Kwa	2·78	0·16-49·11
**Previous studies**	**5·76**	**2·02-16·42**
**Significance: p=0·001, heterogeneity p=0·91**		
**OXVASC and HKU**	**5·76**	**1·22-27·10**
**Significance p=0·027, heterogeneity p=0·20**		
**OVERALL**	**5·81**	**2·63-12·84**
**Significance p<0·0001, heterogeneity p=0·85**		

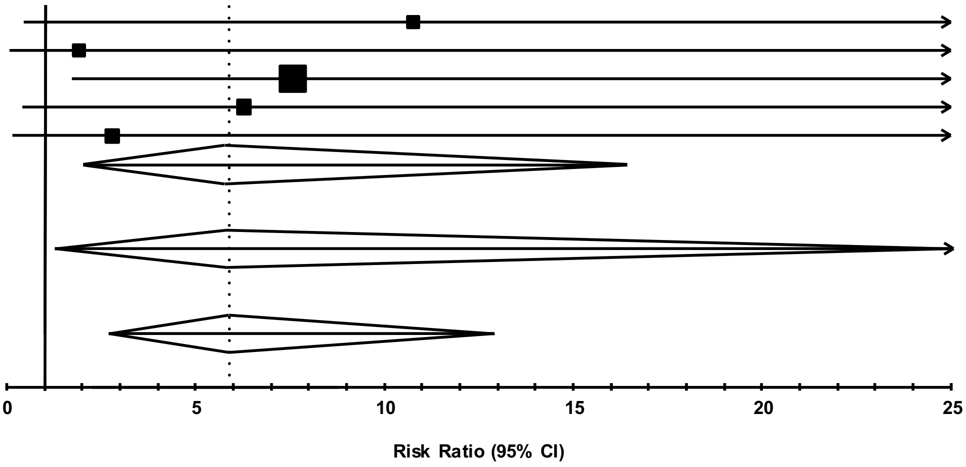

≥5 vs. no microbleeds

Study	RR	95% CI
Huang	44·65	2·43-821·01
Naka	21·83	2·55-187·00
Soo	12·46	3·59-43·19
Thijs	4·13	0·17-99·05
Kwa	14·54	1·90-111·53
**Previous studies**	**14·50**	**6·08-34·62**
**Significance: p<0·0001, heterogeneity p=0·85**		
**OXVASC and HKU**	**11·70**	**3·91-35·30**
**Significance p<0·0001, heterogeneity p=0·46**		
**OVERALL**	**13·35**	**6·75-26·39**
**Significance p<0·0001, heterogeneity p=0·92**		

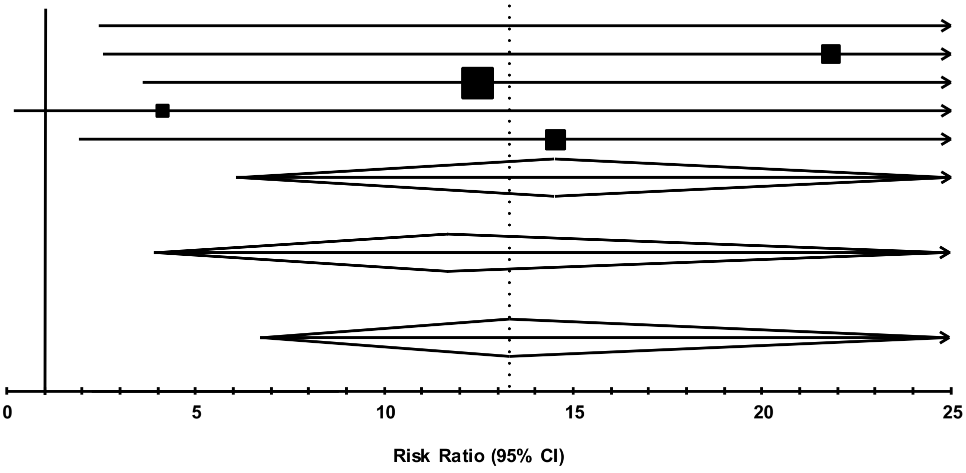

Supplementary Figure V. Pooled analyses of relative risk estimates from the current and previous studies showing risk of recurrent ischaemic stroke (A) and intracerebral hemorrhage (B) in TIA/ischaemic stroke patinets on antiplatelet agents, stratified by presence versus absence of microbleeds

(A) Ischaemic stroke

Study	RR	95% CI
**TIA only cohorts with ≤1 year follow-up**		
Fluri	7·69	1·83-32·40
Lim	4·13	1·83-9·34
**Overall**	**4·80**	**2·36-9·76**
**Significance p<0·0001, heterogeneity p=0·46**		
**Previous TIA/IS cohorts**		
Soo	1·30	0·87-1·94
CROMIS 1	2·68	0·99-7·31
Huang	2·51	1·06-5·97
Fan	1·51	0·49-4·67
Mok	0·85	0·10-7·13
Thijs	1·90	0·97-3·73
Heidelberg	1·30	0·27-6·27
Kwa	2·57	1·06-6·19
Naka	1·01	0·33-3·07
**Overall**	**1·62**	**1·24-2·11**
**Significance p=0·0004, heterogeneity p=0·70**		
**OXVASC and HKU**	**1·56**	**1·15-2·12**
**Significance p=0·005 , heterogeneity p=0·41**		
**All TIA/IS cohorts**	**1·62**	**1·32-1·99**
**Significance p<0·0001, heterogeneity p=0·80**		

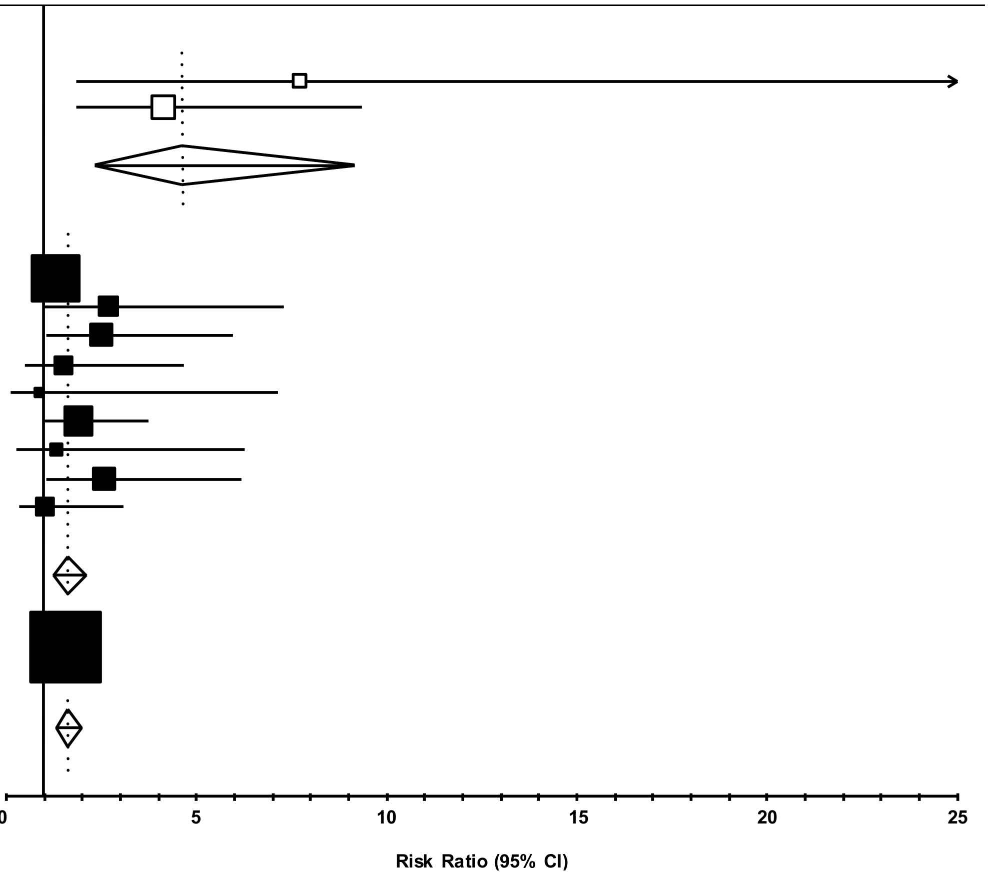

(B) Intracerebral haemorrhage

Study	RR	95% CI
**Previous TIA/IS cohorts**		
Soo	7·16	2·30-22·27
Huang	20·04	1·13-354·24
Fan	7·26	0·84-62·88
Mok	13·65	1·63-114·10
Thijs	2·78	0·17-44·04
Kwa	1·82	0·21-15·93
Naka	12·60	1·51-105·24
**Overall**	**7·09**	**3·43-14·65**
**Significance p<0·0001, heterogeneity p=0·80**		
**OXVASC and HKU**	**4·63**	**1·97-10·85**
**Significance p=0·0004 , heterogeneity p=0·41**		
**All TIA/IS cohorts**	**5·80**	**3·23-10·41**
**Significance p<0·0001, heterogeneity p=0·82**		

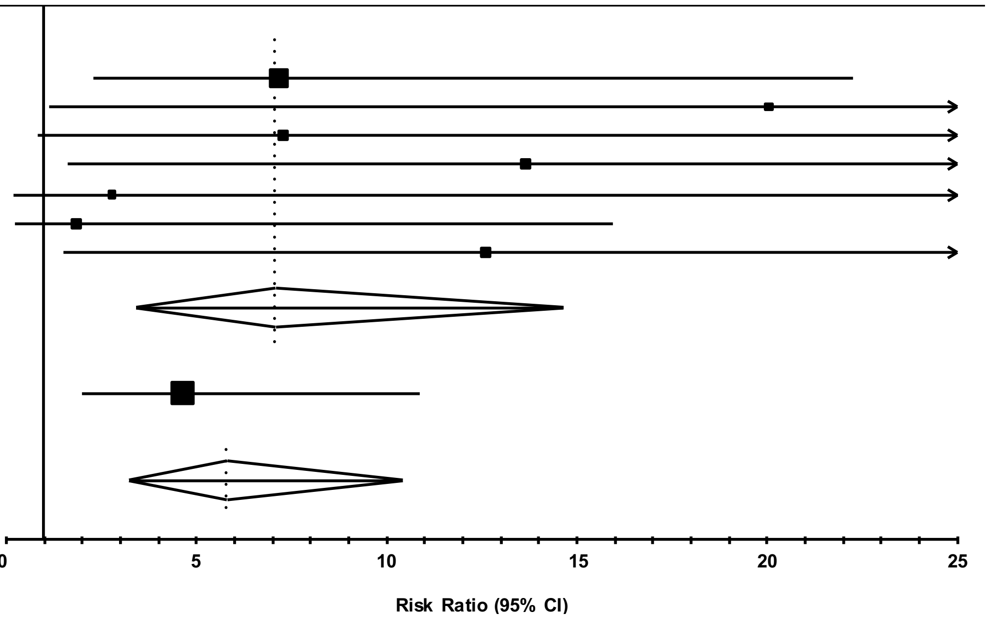

Supplementary Figure VI. Pooled analyses of relative risk estimates from the current and previous studies showing risk of recurrent ischaemic stroke amongst TIA / ischaemic stroke patients with microbleeds versus those without

1 vs. no microbleeds

Study	RR	95% CI
Huang	1·46	0·32-6·73
Imaizumi	2·46	0·24-25·35
Naka	2·18	0·33-14·39
Song	0·45	0·11-1·85
Soo	0·57	0·25-1·28
Fluri	8·82	1·93-40·33
Thijs	1·40	0·49-3·95
Heidelberg	1·60	0·20-12·68
Kwa	2·12	0·66-6·83
Lim	3·09	0·22-42·97
CROMIS 1	3·40	1·17-9·89
**Previous studies**	**1·65**	**0·95-2·86**
**Significance p=0·075, heterogeneity p=0·08**		
**OXVASC and HKU**	**1·55**	**1·05-2·30**
**Significance p=0·029, heterogeneity p=0·62**		
**Overall**	**1·57**	**1·08-2·29**
**Significance p=0·018, heterogeneity p=0·15**		

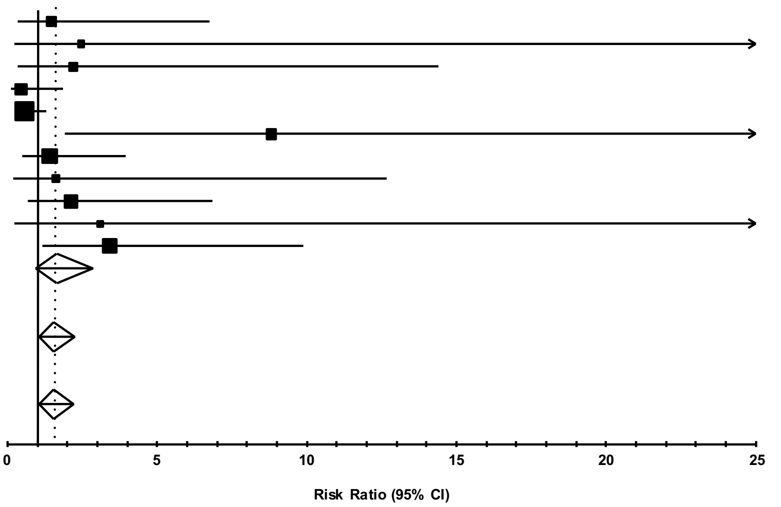

2-4 vs. no microbleeds

Study	RR	95% CI
Huang	2·25	0·75-6·75
Imaizumi	0·67	0·03-13·35
Naka	1·79	0·53-5·98
Song	0·97	0·34-2·72
Soo	2·21	1·31-3·71
Fluri	40·06	13·94-115·13
Thijs	1·65	0·64-4·25
Heidelberg	1·09	0·06-18·39
Kwa	3·16	0·81-12·26
Lim	2·03	0·50-8·27
CROMIS 1	1·70	0·25-11·45
**Previous studies**	**2·47**	**1·28-4·76**
**Significance p=0·007, heterogeneity p=0·0003**		
**OXVASC and HKU**	**1·37**	**0·85-2·21**
**Significance p=0·19, heterogeneity p=0·77**		
**OVERALL**	**2·19**	**1·29-3·70**
**Significance p=0·004, heterogeneity p=0·0002**		

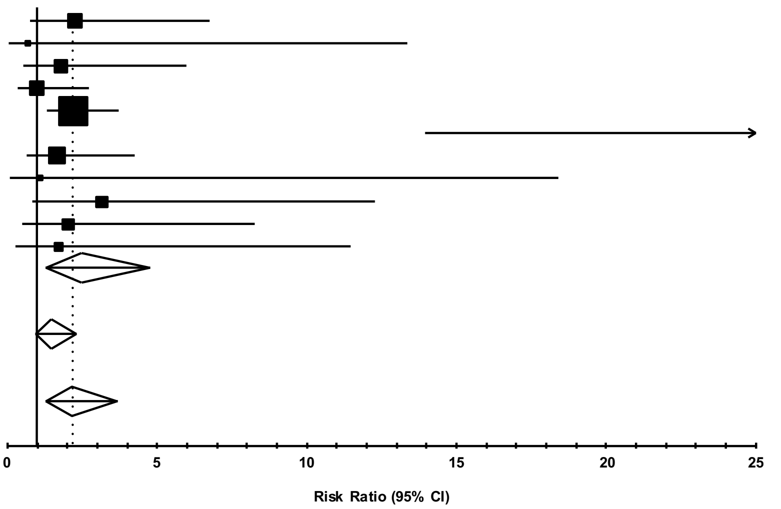

≥5 vs. no microbleeds

Study	RR	95% CI
Huang	3·76	1·34-10·53
Imaizumi	1·97	0·29-13·41
Naka	0·25	0·02-4·15
Song	0·32	0·02-5·18
Soo	1·56	0·88-2·75
Fluri	16·67	2·36-117·55
Thijs	2·69	0·98-7·37
Heidelberg	1·95	0·25-15·35
Kwa	3·42	0·54-21·72
Lim	8·46	3·63-19·74
CROMIS 1	2·13	0·33-13·60
**Previous studies**	**2·75**	**1·53-4·95**
**Significance p=0·0007, heterogeneity p=0·030**		
**OXVASC and HKU**	**1·92**	**1·24-2·97**
**Significance p=0·004, heterogeneity p=0·41**		
**OVERALL**	**2·53**	**1·64-3·90**
**Significance p<0·0001, heterogeneity p=0·038**		

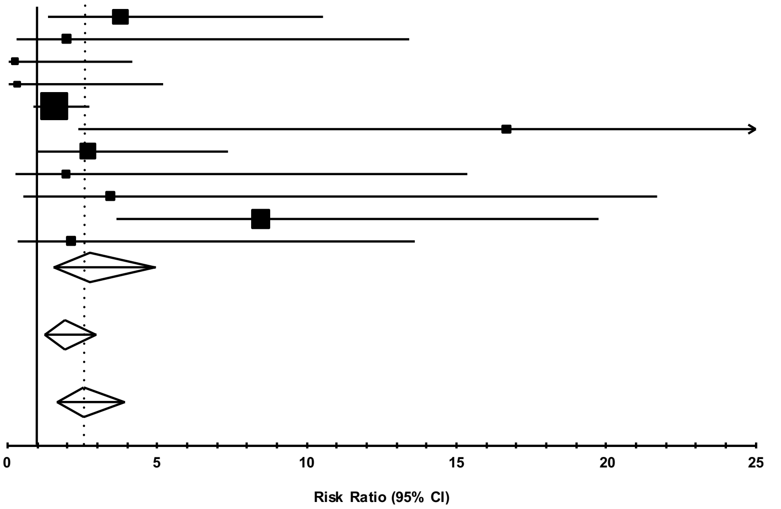

Supplementary Figure VII. Pooled analyses of relative risk estimates from the current and previous studies showing risk of intracerebral haemorrhage amongst TIA / ischaemic stroke patients with microbleeds versus those without

1 vs. no microbleeds

Study	RR	95% CI
Huang	17·33	0·71-420·91
Imaizumi	14.00	0·60-327·25
Naka	21·83	1·55-308·50
Song	3·67	0·84-16·08
Soo	3·04	0·56-16·38
Thijs	2·18	0·09-52·74
Kwa	1·30	0·07-23·51
**Previous studies**	**4·57**	**1·94-10·74**
**Significance p=0·0005, heterogeneity p=0·71**		
**OXVASC and HKU**	**1·18**	**0·26-5·38**
**Significance p=0·83, heterogeneity p=0·38**		
**OVERALL**	**3·30**	**1·57-6·95**
**Significance p=0·002, heterogeneity p=0·56**		

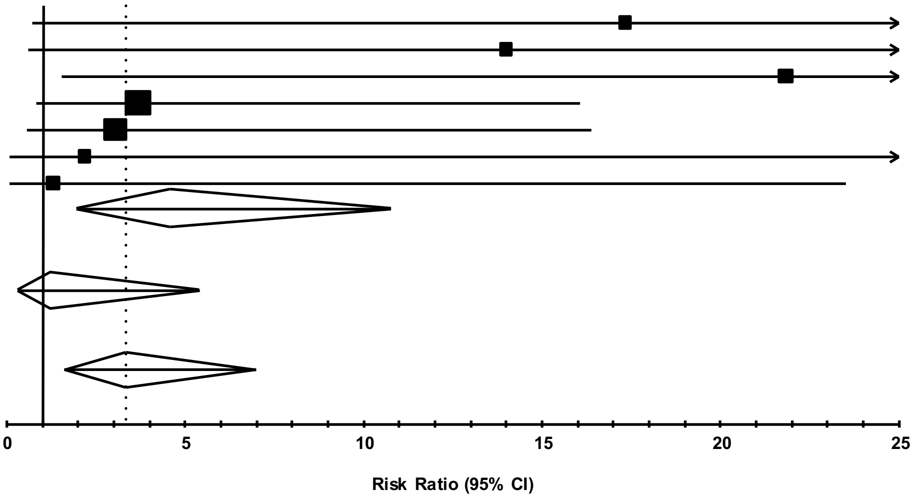

2-4 vs. no microbleeds

Study	RR	95% CI
Huang	10·75	0·44-262·01
Imaizumi	16·67	0·83-333·74
Naka	1·91	0·08-45·54
Song	1·33	0·15-11·70
Soo	7·57	1·73-33·09
Thijs	6·28	0·40-99·00
Kwa	2·78	0·16-49·11
**Previous studies**	**4·93**	**2·00-12·14**
**Significance p=0·0005, heterogeneity p=0·80**		
**OXVASC and HKU**	**5·21**	**1·02-26·60**
**Significance p=0·047, heterogeneity p=0·11**		
**OVERALL**	**5·07**	**2·60-9·88**
**Significance p<0·0001, heterogeneity p=0·67**		

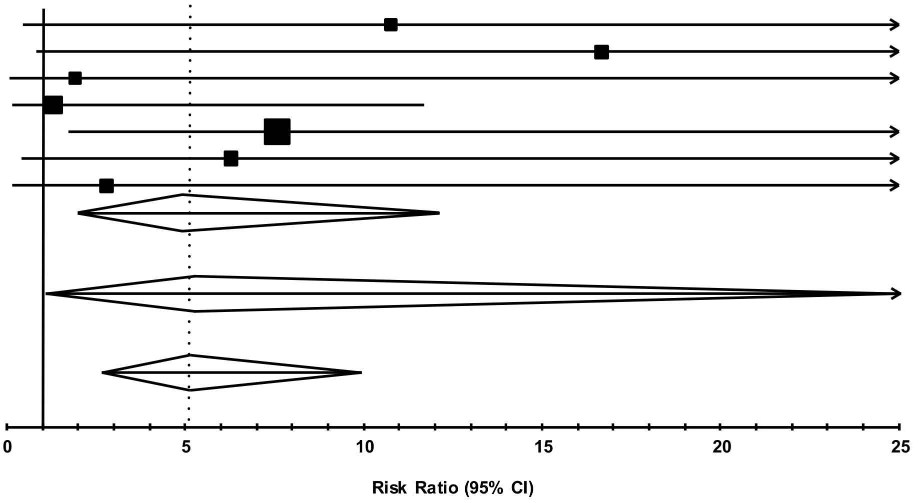

≥5 vs. no microbleeds

Study	RR	95% CI
Huang	44·65	2·43-821·01
Imaizumi	25·28	1·46-436·22
Naka	21·83	2·55-187·00
Song	11·31	2·68-47·79
Soo	12·46	3·59-43·19
Thijs	4·13	0·17-99·05
Kwa	14·54	1·90-111·53
**Previous studies**	**14·12**	**6·87-29·02**
**Significance p<0·0001, heterogeneity p=0·95**		
**OXVASC and HKU**	**8·75**	**3·44-22·24**
**Significance p<0·0001, heterogeneity p=0·51**		
**OVERALL**	**11·81**	**6·68-20·88**
**Significance p<0·0001, heterogeneity p=0·95**		

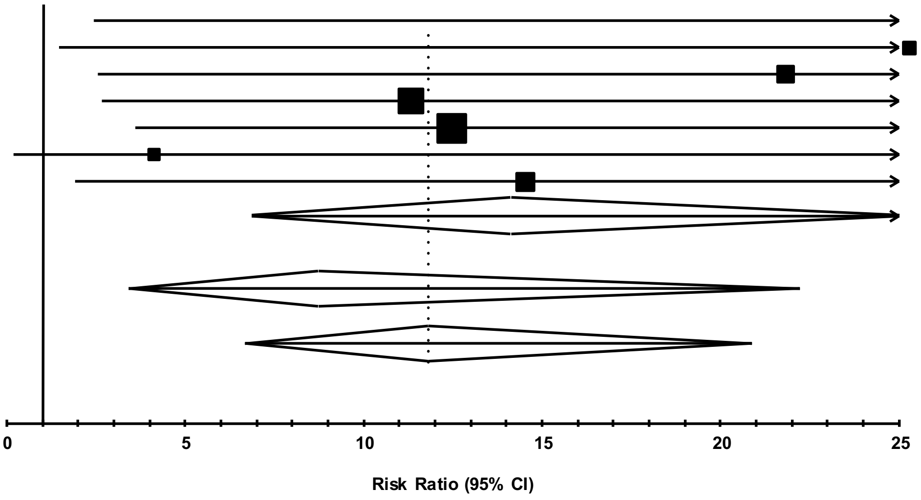

Supplementary references

1. Anderson JL, Adams CD, Antman EM, Bridges CR, Califf RM, Casey DE, Jr., et al. 2012 accf/aha focused update incorporated into the accf/aha 2007 guidelines for the management of patients with unstable angina/non-st-elevation myocardial infarction: A report of the american college of cardiology foundation/american heart association task force on practice guidelines. *Circulation*. 2013;127:e663-828
2. O'Gara PT, Kushner FG, Ascheim DD, Casey DE, Jr., Chung MK, de Lemos JA, et al. 2013 accf/aha guideline for the management of st-elevation myocardial infarction: Executive summary: A report of the american college of cardiology foundation/american heart association task force on practice guidelines. *Circulation*. 2013;127:529-555
3. Mehta SR, Yusuf S, Peters RJ, Bertrand ME, Lewis BS, Natarajan MK, et al. Effects of pretreatment with clopidogrel and aspirin followed by long-term therapy in patients undergoing percutaneous coronary intervention: The pci-cure study. *Lancet*. 2001;358:527-533
4. Kwa VI, Algra A, Brundel M, Bouvy W, Kappelle LJ, Group MS. Microbleeds as a predictor of intracerebral haemorrhage and ischaemic stroke after a tia or minor ischaemic stroke: A cohort study. *BMJ Open*. 2013;3
5. Fluri F, Jax F, Amort M, Wetzel SG, Lyrer PA, Katan M, et al. Significance of microbleeds in patients with transient ischaemic attack. *European journal of neurology : the official journal of the European Federation of Neurological Societies*. 2012;19:522-524
6. Thijs V, Lemmens R, Schoofs C, Gorner A, Van Damme P, Schrooten M, et al. Microbleeds and the risk of recurrent stroke. *Stroke; a journal of cerebral circulation*. 2010;41:2005-2009
7. Boulanger JM, Coutts SB, Eliasziw M, Gagnon AJ, Simon JE, Subramaniam S, et al. Cerebral microhemorrhages predict new disabling or fatal strokes in patients with acute ischemic stroke or transient ischemic attack. *Stroke; a journal of cerebral circulation*. 2006;37:911-914
8. Lau KK, Wong YK, Teo KC, Chang RSK, Tse MY, Hoi CP, et al. Long-term prognostic implications of cerebral microbleeds in chinese patients with ischemic stroke. *J Am Heart Assoc*. 2017;6
9. Lim JS, Hong KS, Kim GM, Bang OY, Bae HJ, Kwon HM, et al. Cerebral microbleeds and early recurrent stroke after transient ischemic attack results from the korean transient ischemic attack expression registry. *Jama Neurol*. 2015;72:301-308
10. Song TJ, Kim J, Lee HS, Nam CM, Nam HS, Heo JH, et al. The frequency of cerebral microbleeds increases with chads(2) scores in stroke patients with non-valvular atrial fibrillation. *European journal of neurology : the official journal of the European Federation of Neurological Societies*. 2013;20:502-508
11. Mok VC, Lau AY, Wong A, Lam WW, Chan A, Leung H, et al. Long-term prognosis of chinese patients with a lacunar infarct associated with small vessel disease: A five-year longitudinal study. *International journal of stroke : official journal of the International Stroke Society*. 2009;4:81-88
12. Soo YO, Yang SR, Lam WW, Wong A, Fan YH, Leung HH, et al. Risk vs benefit of anti-thrombotic therapy in ischaemic stroke patients with cerebral microbleeds. *Journal of neurology*. 2008;255:1679-1686
13. Huang Y, Cheng Y, Wu J, Li Y, Xu E, Hong Z, et al. Cilostazol as an alternative to aspirin after ischaemic stroke: A randomised, double-blind, pilot study. *The Lancet. Neurology*. 2008;7:494-499
14. Naka H, Nomura E, Takahashi T, Wakabayashi S, Mimori Y, Kajikawa H, et al. Combinations of the presence or absence of cerebral microbleeds and advanced white matter hyperintensity as predictors of subsequent stroke types. *AJNR. American journal of neuroradiology*. 2006;27:830-835
15. Fan YH, Zhang L, Lam WW, Mok VC, Wong KS. Cerebral microbleeds as a risk factor for subsequent intracerebral hemorrhages among patients with acute ischemic stroke. *Stroke; a journal of cerebral circulation*. 2003;34:2459-2462
16. Imaizumi T, Horita Y, Hashimoto Y, Niwa J. Dotlike hemosiderin spots on t2*-weighted magnetic resonance imaging as a predictor of stroke recurrence: A prospective study. *Journal of neurosurgery*. 2004;101:915-920
